# Supplementary material for: Protocol for the INFORMED (Individualised Patient Care and Treatment for Maternal Diabetes) Study: a randomised controlled trial embedded within routine care
Source: BMJ Open. 2023 Feb 27;13(2):e065388. doi: 10.1136/bmjopen-2022-065388 (PMC9972421; doi:10.1136/bmjopen-2022-065388)
Supplement: Supplementary data [file bmjopen-2022-065388supp001.pdf]

UNIVERSITY OF LEEDS

NHS
The Leeds
Teaching Hospitals
NHS Trust

# INFORMED

Individualised patieNt care and treatment FOR MatErnal Diabetes

## SUPPLEMENTARY MATERIALS

### Supplementary section: A

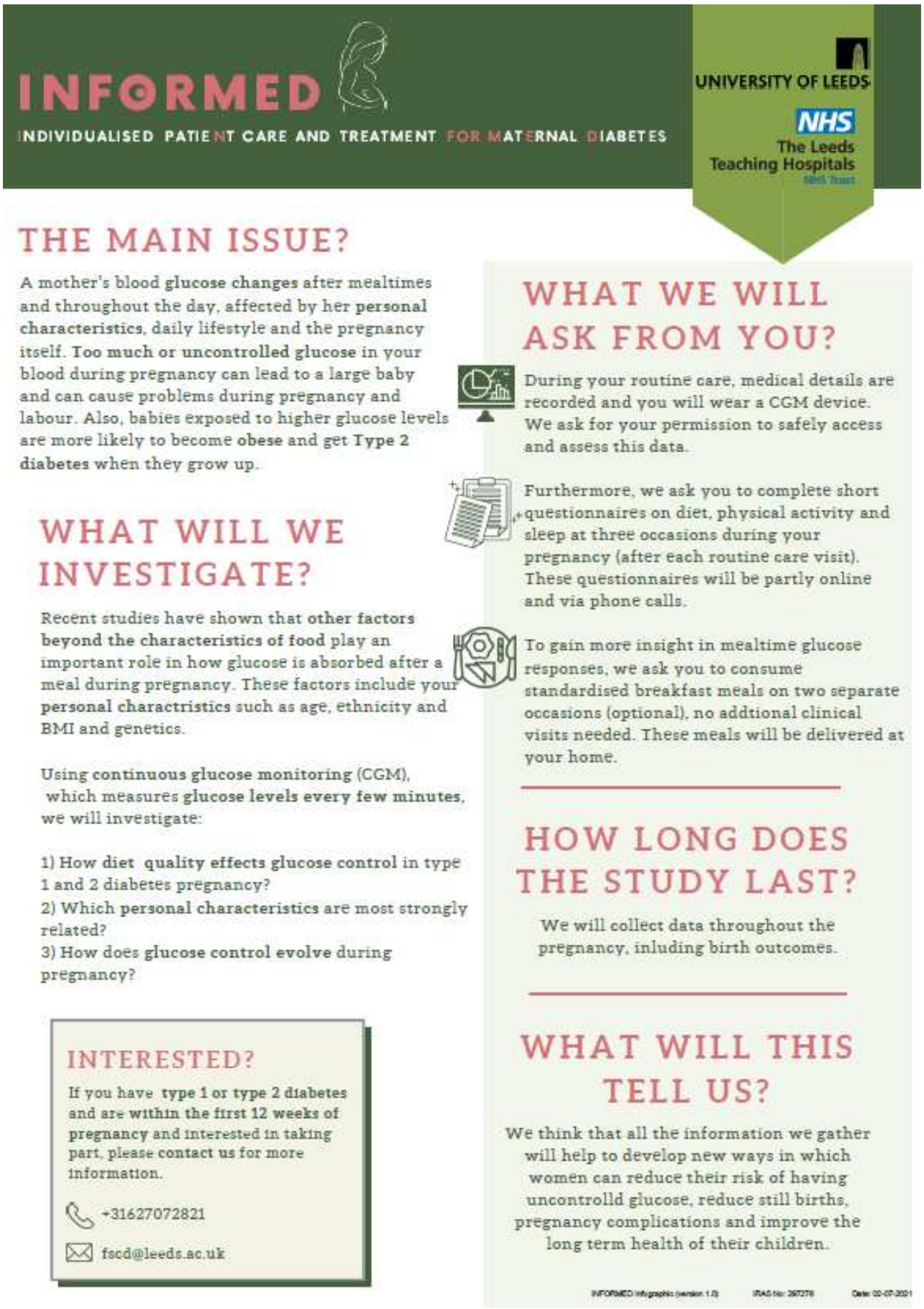


INFORMED Protocol (version 1.0)   IRAS No: 297276   Date: 22-07-2021

UNIVERSITY OF LEEDS

NHS
The Leeds Teaching Hospitals
NHS Trust

# Supplementary section B: Participant Information Sheet

INFORMED

Individualised patieNt care and treatment FOR MatErnal Diabetes

**Understanding the glycaemic profile of maternal diabetes using continuous glucose monitoring: intensive glucose profiling to inform patient care and treatment**

## Participant Information Sheet

Thank you for considering your participation in our study called INFORMED, which is part of a PhD research project at the School of Food Science and Nutrition. We - as research team - would like to provide you with details about the study, what your role will involve, and other key information before you decide.

Please ask us (*contact details at the end of the handout*) if there is anything that is not clear or you would like more information. Take time to decide whether or not you wish to take part.

**Study information**

**What is the purpose of the study?**

During pregnancy, a mother's blood glucose level changes constantly across 24-hour period, and is affected by her physical characteristics, lifestyle, and the pregnancy itself. While many factors affect the way babies grow in the womb, one of the easiest to measure and modify, is the amount of glucose that they get from their mother. Uncontrolled or too much glucose in their mother's blood during pregnancy, usually leads to a large baby and can increase the chance of problems during pregnancy, labour, and immediately after birth for both mother and child. Being born too small can also be problematic and has been linked to increases the chances of obesity and type 2 diabetes.

Glucose levels of the mother rise after meal consumption and, if uncontrolled, can contribute to some of these health concerns. While the type of food being eaten is vital, recent studies have shown that other factors (such as age, ethnicity, activity levels, and sleep duration) also play a part. However, despite knowing these factors, we currently do not know how to modify a meal to match a mother's characteristics and how a mother's diet effects glucose levels throughout pregnancy. As part of your routine care you are wearing a continuous glucose monitor. With this study we are investigating the impact of diet and lifestyle affects on glucose control throughout pregnancy as there is currently very little information on how diet and lifestyle affects glucose levels measures during pregnancy.

Therefore, as a first step, we want to monitor and study how 24-hour and mealtime glucose levels change in response to diet and across pregnancy in women with pre-existing type 1 or type 2 diabetes. Most information on glucose control and medical data will be requested via your medical records, if you give us permission for us to access your data. To decrease the burden of participation, we will use existing data as much as possible. However, to be able assess your diet and lifestyle during pregnancy, we will ask you to complete questionnaires via phone calls. These questionnaires are detailed below. None of the information obtained via the questionnaires will be shared with your clinical care team. This data on lifestyle will be anonymised and is solely for the purpose of this study.



Dingena CF, *et al. BMJ Open* 2023; 13:e065388. doi: 10.1136/bmjopen-2022-065388

UNIVERSITY OF LEEDS

NHS The Leeds Teaching Hospitals NHS Trust

**Why have I been invited?**
We are approaching women with type 1 and type 2 diabetes, who are early in their pregnancy, to help us with this study. You do not have to take part – it is completely up to you — and does not affect the quality of care you receive from the NHS. Also, if you choose to take part and later decide to withdraw (for any or no reason), it will not affect your quality of care from the NHS.

**What could my participation do?**
By taking part in this study, you will help us to better understand how glucose levels change during pregnancy in women with type-1 and type-2 diabetes and the role of diet. By knowing this, we can design special diets and nutritional strategies to minimise the chances of babies being exposed to abnormal glucose levels and their risk of future health problems.

**What would taking part involve?**
*Medical information*. We ask you to give us consent to access selected parts of your medical record that reflect your general health and the health of your pregnancy (e.g., blood pressure, blood/urine test results, current medication, diabetes related pregnancy outcomes) and your diabetes health risks (e.g., age, body weight, ethnicity). Additionally, once you have given birth, your baby's birthweight, and any pregnancy complications will be copied from your medical records.

*Urine samples*. You will be providing urine samples regularly during pregnancy to your clinical team. Once they have been tested, rather than throwing them away we would like your consent to keep the remaining sample for future metabolic analysis.

*Blood Samples*. You will be having blood taken regularly during this pregnancy for your clinical care. On three of your routine visits to the Diabetes in Pregnancy Clinic we would like your consent to take an additional 10ml of blood for the study. We will store this to look at molecular and genetic markers that may be involved in metabolism and diabetes later. No infant blood samples are requested.

All samples will be stored at the University of Leeds in designated Human Tissue Act approved and compliant facilities.

*Glucose Data*. We ask for your consent for us to access your clinical glucose data throughout pregnancy. This will require no additional work on your part.

*Lifestyle Questionnaires*. On three occasions during your pregnancy, at ~10-12, ~18-20, and ~28-34, we will contact you at your convenience by phone or video call to complete some short questionnaires about your habitual physical activity, sleep quality / patterns and mealtimes. Also, following these three clinical visits, we will ask you to keep track of your diet for 3 days (2 weekdays and 1 weekend day) using an online dietary tracker called MyFood24. During the phone call, we will explain you how to use this dietary tracker. The phone calls will last not more than 30 minutes. The dietary tracker will take approximately 10 minutes per day to complete. This data will be anonymised and none of this data will be shared with your clinical care team.

*Breakfast replacements*. To gain more insight into mealtime glucose responses, we would like you to consider taking part in additional part of the study, where we will provide you with two different breakfast shakes to drink instead of your usual breakfast for 4 days on two separate occasions (one during your 2nd and one during your 3rd trimester). The two different breakfast shakes (e.g. Shake 1 and Shake 2) have the same amount of carbohydrate as that recommended during pregnancy, but one is designed to be absorbed slower, and the other faster so we can see how this affects your glucose measures on the continuous glucose monitor. Dependent on your randomization you will consume Shake 1 for two days followed by Shake 2 or vice versa. They are vegan friendly. The shakes will be delivered to your home with instructions for you to prepare.

You can of course still participate in the main study without having to take the breakfast shakes if you prefer.

This study will tell us, in detail previously unseen, how (mealtime) glucose changes across pregnancy and how diet can best be used to manage glucose levels and minimise maternal and infant health risks.

**What are the possible risks of taking part?**
Although we have designed the meals to not contain allergens and to release the same amount of glucose as you would usually eat for breakfast there is a possibility that you may experience an allergic reaction or higher glucose levels than normal after the standardised meal consumption. We will check that you have no allergies before taking part and ask you to contact the research team if you have these reactions to the meal. You will be advised to monitor and manage your blood glucose levels like you normally would and feel most comfortable with. However, if blood glucose levels surpass >18mmol/L for more than 90-minutes you are advised to administer a corrective dose of insulin or contact your GP/clinical care team. The meals are designed to minimize risk of hyperglycaemia. Blood samples are part of your routine clinical care and will be performed by qualified clinical staff, so any discomfort should be minimal.

**What are the possible benefits of taking part?**
There are no specific benefits to you of taking part, but participating in this study will give us important information about how to assess glucose in relation to personal characteristics, pregnancy outcomes, and newborn health. We anticipate that this will then help us to identify and develop new diet strategies to help women reduce their risk of small or large babies, stillbirths, pregnancy complications, and improve the long-term health of their children.

**Further Information**

**What will happen if I don't want to carry on with the study?**
You are free to withdraw at any time without explanation. If you decide not to carry on, it will not affect your care in anyway.

**What if something goes wrong?**
During the study, you will be covered by the Sponsor's Insurance, the University of Leeds is acting as Sponsor for this study. The University of Leeds has insurance cover in force, which meets claims against it and where those claims arise from the Universities own negligence in its role and activities relating to the study (and which is subject to the terms, conditions and exceptions of the relevant policy). Clinical negligence indemnification will rest with the participating NHS Trust under standard NHS arrangements.

If you are unhappy about any part of the study, you are encouraged to discuss this with the research team or with the Patient Assistance and Liaison Services (PALS) at your hospital. Normal legal processes are also open to you. We foresee minimal risks as most data will be collected from your routine clinical records and specific study risks are limited to questionnaires and meal replacements.

**What will happen to my additional blood samples and urine sample?**
Your blood and urine samples will be labelled with your unique study number and stored in freezers at the University of Leeds for longer term storage. Analysis of the samples will be undertaken for molecular and genetic factors that may contribute to a mother's metabolism, glucose control and babies growth. Only researchers directly involved in the study will have access to the samples. Results of samples analyses will only be used for the purpose of the research study.

**How will we use information about you?**
We will need to use information from you and from your medical records (including the continuous glucose monitoring data) for this research project. This information will include your initials/NHS number/date of birth/name/contact details. A member of your clinical care team will give you a unique random study

UNIVERSITY OF LEEDS

NHS
The Leeds Teaching Hospitals NHS Trust

number, so that personal information cannot be traced back by members of the research team. Your consented medical data will be routinely stored on an electronic patient platform using your study number. Only authorised members of the research team will be able to access this platform and copy the data to secure university computers, this data will be stored for a maximum of 15 years, for analysis and writing up the results and also for authorised people to check your records to make sure that the research is being done properly. These people who do not need to know who you are will not be able to see your name or contact details. Your data will have a unique study number that will make you anonymous to the study team. We will keep all information about you safe and secure. Once we have finished the study, we will keep some of the data so we can check the results. All analyses and reports will be written in a way that no-one can work out that you took part in the study.

**How will my information be kept confidential?**
All information which is collected about you will be held securely and treated in accordance with the Regulation (EU) 2016/679 (General Data Protection Regulation) and the Data Protection Act 2018.

We will be using information collected by your local hospital from you and your medical records in order to undertake this study. No personal identifiable data will leave the NHS hospital without your consent; Data leaving the hospital will be labelled with your unique study number and will not have your name or any other identifying details on it. We refer to this as linked anonymised data as it is linked to you by a code. The code will only be known by key research team members. It will be kept securely.

Data which leaves the NHS Trust where you are being treated will be held securely in a database, operated by the data analysis team at the University of Leeds. This includes only linked anonymised study data and will not have your name or any other identifying details on it.

If you join the study, the data collected for the study, together with any relevant medical records, may be looked at by authorised persons from University of Leeds, the Research and Development Department of your local hospital and the Regulatory authorities to check the study is being carried out correctly. They all have a duty of confidentiality to you as a research participant.

Other third party researchers (e.g. universities, NHS organisations or companies involved in health and care research) may wish to access anonymised data (including samples) from this study in the future (anonymised data do not include names, addresses, or dates of birth, and it is not possible to identify individual participants from anonymised data). If this is the case, the Chief Investigator will ensure that the other researchers comply with legal, data protection and ethical guidelines. This may include research outside of the UK and EU and/or research that is commercial in nature. Your data will be stored securely for a period of 15 years after the end of the trial before being destroyed.

**What are your choices about how your information is used?**
If you withdraw consent during the study, no further data will be collected on you. However, any data (including samples) already collected by the research team may be retained and subsequently analysed for the purposes of the study. Your right to access, change or move your information are limited, as we need to manage your information in specific ways in order for the research to be reliable and accurate. To safeguard your rights we will use the minimum personally-identifiable information possible.

The University of Leeds as the Sponsor, is the data controller for this study. This means that we are responsible for looking after your information and using it properly.

The University of Leeds is the data processors for this study. The lawful basis for processing personal data collected in this study is that it is a task in the public interest. You can find out more about how we use your information at https://dataprotection.leeds.ac.uk/wp-content/uploads/sites/48/2019/02/Research-Privacy-Notice.pdf; and https://dataprotection.leeds.ac.uk/wp-content/uploads/sites/48/2019/09/HRA-



Dingena CF, *et al. BMJ Open* 2023; 13:e065388. doi: 10.1136/bmjopen-2022-065388

UNIVERSITY OF LEEDS

The Leeds Teaching Hospitals NHS Trust

transparency-wording.pdf by contacting University of Leeds Data Protection Officer's (e-mail: dpo@leeds.ac.uk).

**What will happen to the study results?**
The study is part of a PhD project and the result will be used for writing the doctorate thesis. The study results may be presented at meetings or published in scientific journals but individuals will not be identifiable. After the study has ended we will send a newsletter with the study results to your research team, which they will be able to share with you.

**Who is organising and funding the research?**
The research study has been primarily funded and sponsored by the University of Leeds with additional support from the Wellcome Trust (MZ). The Chief Investigators (Dr Michael Zulyniak and Professor Eleanor Scott) are University of Leeds researchers. Professor Scott is also one of the senior NHS consultants providing clinical care in the Diabetes Pregnancy Clinic.

**Who has reviewed the study?**
Before any research goes ahead it has to be checked by an Ethics Committee. This study has been reviewed by the Leeds East Research Ethics Committee.

**What happens now if I agree to do the study?**
The study procedures will be explained to you in more detail by the research team. You will be able to ask questions and voice any queries. If you agree to take part we will ask you to sign a consent form and complete screening questionnaires online to confirm eligibility, this will take approximately 10 minutes. If you are not eligible to participate, information provided prior to participation will be destroyed. The research team will then co-ordinate with you the dates for completing the lifestyle questionnaires, food dairy, and consuming the breakfast replacements

**Please, contact the research team for more information:**


*Co-Investigator*

Name: Cassy Dingena
Address: EC Stoner Building, University of Leeds, Woodhouse Ln, LS2 9JT
Email: fscd@leeds.ac.uk
Telephone: +316 27072821

*Chief Investigators*

Name: Dr Michael Zulyniak
Address: EC Stoner Building, University of Leeds, Woodhouse Ln, LS2 9JT
Email: m.a.zulyniak@leeds.ac.uk
Telephone: +44 (0)113 343 0685

Name: Professor Eleanor Scott
Address: Manny Cussins Diabetes Centre, St James's University Hospital, Leeds, LS9 7TF
Email: eleanor.scott9@nhs.net
Telephone 0113 2065014

UNIVERSITY OF LEEDS

NHS The Leeds Teaching Hospitals NHS Trust

# Supplementary section C: Consent form

INFORMED

Individualised patieNt care and treatment FOR MatErnal Diabetes

**Understanding the glycaemic profile of maternal diabetes using continuous glucose monitoring: intensive glucose profiling to inform patient care and treatment**

**IRAS Project ID: 297276**

**Participant ID for this study:**

**Name of Researcher**:

## CONSENT FORM

Please **initial** all boxes that apply:

1. I confirm that I have read and understand the Participant Information Sheet dated ………………………. (Version……….) for the above study. I have had the opportunity to consider the information, ask questions and have had these answered satisfactorily. ☐

2. I understand that my participation is voluntary and that I am free to withdraw at any time without giving any reason, without my medical care or legal rights being affected in any way. ☐

3. I understand that relevant sections of my medical records of me and my baby, after their birth until either of us is discharged from hospital and data collected during this study may be looked at by members of the research team, from regulatory authorities or from the NHS Trust / sponsor, in this case the University of Leeds, where it is relevant to taking part in this research. I give permission for these individuals to have access to my records. ☐

4. I understand that my name will not be linked with the research materials, and I will not be identified or identifiable in the report or reports that result from the research. ☐

5. I agree for any unused urine (up to 2.5 mL) collected as routine care during this study to be stored securely long-term at University of Leeds for later analysis for research purposes only. These are considered a 'gift' from me, and may be used in relevant future research (in an anonymised form). I understand that this may involve co-operation with researchers outside of the UK) (*optional*). ☐

INFORMED Protocol (version 1.0)    IRAS No: 297276    Date: 22-07-2021

UNIVERSITY OF LEEDS

The Leeds Teaching Hospitals NHS Trust

6. I agree for any additional blood samples collected during this study to be stored securely long-term at University of Leeds for later analysis for research purposes only. These are considered a 'gift' from me, and may be used in relevant future research (in an anonymised form). I understand that this may involve co-operation with researchers outside of the UK) (*optional*). ☐

    a. I give my consent for the taking of an additional blood sample for molecular analysis (*optional*). ☐

    b. I give my consent for the taking of an additional blood sample for genetic/DNA analysis (*optional*). ☐

7. I understand that the information collected about me and my baby may be used to support other ethically approved research in the future, and may be shared anonymously with other researchers. This may include research outside of the UK and EU and/or research that is commercial in nature. ☐

8. I agree for my GP to be informed of my participation in this study. ☐

9. I give consent to the research team to keep my contact details for them to contact me during and after the study (*optional*). ☐

10. I am happy to be contacted about longer term follow up after ending of the study of myself or my baby (*optional*). ☐

11. I agree to take part in the standardised meals study (*optional*). ☐

12. I agree to take part in the study to the sections I have consented to. ☐

| Name of participant | Date | Signature |
|---|---|---|
| Name of person taking consent | Date* | Signature* |

*To be signed and dated in the presence of the participant.

INFORMED Protocol (version 1.0) IRAS No: 297276 Date: 22-07-2021

UNIVERSITY OF LEEDS

NHS
The Leeds Teaching Hospitals
NHS Trust

# Suplementary section D: Screening Questionnaire

INFORMED

Individualised patieNt care and treatment FOR MatErnal Diabetes

**Understanding the glycaemic profile of maternal diabetes using continuous glucose monitoring: intensive glucose profiling to inform patient care and treatment**

## Screening questionnaire

Thank you for considering your participation in our study called PERFORMD. We would like to fill out this questionnaire, so we can assess your eligibility.

First name/s: …………………………………………………………………………………………………

Last name: ………………………………………………………………………………………………

Address: ………………………………………………………………………………………………

Postcode:………………………………………………………………………………………………

Phone number:………………………………………………………………………………………

E-mail address:……………………………………………………………………………………

Date of birth: …………………………………………………………………………………………

**General Practitioner (GP)/Family doctor**

Name:…………………………………………………………………………………………………

Address:………………………………………………………………………………………………

Postcode:………………………………………………………………………………………………

Phone number:………………………………………………………………………………………

UNIVERSITY OF LEEDS

NHS The Leeds Teaching Hospitals NHS Trust

**Medical specialist (if applicable)**

Name: ………………………………………………………………………………………….........

Address:……………………………………………………………………………………….........

Postcode: ……………………….………………………………………………………...........

Phone number: ……………………………………………………………………………...........

1. What is your ethnic origin?
   - ☐ White (Caucasian)
   - ☐ Black (African-American)
   - ☐ Asian
   - ☐ Mixed

2. What is the highest educational qualification that you have?
   - ☐ No qualifications
   - ☐ Achieved GCSE grades D-G, NVQ Level 1, Skills For Life level 1, BTEC-award Certificate or diploma level 1, OCR National
   - ☐ GCSE grades A*-C, NVQ Level 2, BTEC Award Certificate OR diploma level 2
   - ☐ AS & A level, NVQ Level 3, Advanced Extension award, International Baccalaureate, OCR National
   - ☐ NVQ Level 4, BTEC Professional award, Certificate of Higher Education
   - ☐ BTEC Award Advanced professional / Bachelors Degree / Graduate Diploma
   - ☐ University Masters Degree / Postgraduate diploma / NVQ Level 5 / BTEC Advanced Professional Award Certificate and Diploma level 7
   - ☐ Doctorate (e.g. PhD, DClin.)



Dingena CF, *et al. BMJ Open* 2023; 13:e065388. doi: 10.1136/bmjopen-2022-065388

UNIVERSITY OF LEEDS

NHS The Leeds Teaching Hospitals NHS Trust

3. What do you do?
☐ I am a student
☐ I am employed
☐ I am self employed
☐ I am a housewife, househusband
☐ I am unemployed
☐ I am unable to work (e.g. due to a disability)
☐ I am retired
☐ I do something else (e.g. volunteering), namely.........................................................................................................

4. When is your expected due date?
*If you do not know exactly, try to estimate it as well as possible.*

|__|__| |__|__| |__|__|__|__|
Day Month Year

5. How long have you been pregnant? (weeks)
*If you do not know exactly, try to estimate it as well as possible.*

………………………………

6. Do you have children?
☐ Yes, I have …. child(ren).
☐ No

7. Do you have a singleton pregnancy?
☐ Yes, I am expecting an single child
☐ No, I am expecting twins, triplets etc.

8. Would you say your general health is…..?
☐ Excellent
☐ Very Good
☐ Good
☐ Fair
☐ Poor
☐ Don't know/Not sure
☐ I rather not say

9. Are you diagnosed with diabetes (Type 1, Type 2, Gestational diabetes)?
☐ Yes, Please specify type of diabetes ……………………………
☐ No

10. For how long have you been diagnosed with diabetes?
*If you do not know exactly, try to estimate it as well as possible.*

Please, specify in years ..........

11. Did you take any medication (including diabetes medication) in the last month?
☐ No
☐ Yes

If yes, please specify which medication, the dose and how many times a day.

For example: Routine: Omeprazole 40mg once a day for 7 days or one-time treatment: amoxicillin 500mg once a for 7 days.

.......................................................................................................................

.......................................................................................................................

.......................................................................................................................

.......................................................................................................................

.......................................................................................................................

12. Do you currently smoke or use e-cigarettes?
☐ Yes - smoke cigarettes
☐ Yes - smoke cigars
☐ Yes - use e- cigarettes
☐ No, I quit smoking → go on to question 22
☐ No, I never smoked → go on to question 23

13. How many cigarettes/cigars do you normally smoke?
☐ 1-5 each day
☐ 6-10 each day
☐ More than 10 each day
☐ None, I smoke pipe or vape

14. What year did you start and quit smoking?
Started: (yyyy) |__|__|__|__|
Quit: (yyyy) |__|__|__|__|

UNIVERSITY OF LEEDS

NHS
The Leeds Teaching Hospitals
NHS Trust

15. How many units of alcohol did you normally (before pregnancy) consume during the week? (A Guide to the number of units of alcohol in some typical alcoholic drinks is provided). https://www.nhs.uk/Livewell/alcohol/Pages/alcohol-units.aspx

☐ I do not use any alcohol
☐ Less than 1 unit a week
☐ 1 - 5 units a week
☐ 6 - 7 units a week
☐ 8 - 15 units a week
☐ 16 - 30 units a week
☐ More than 30 units a week

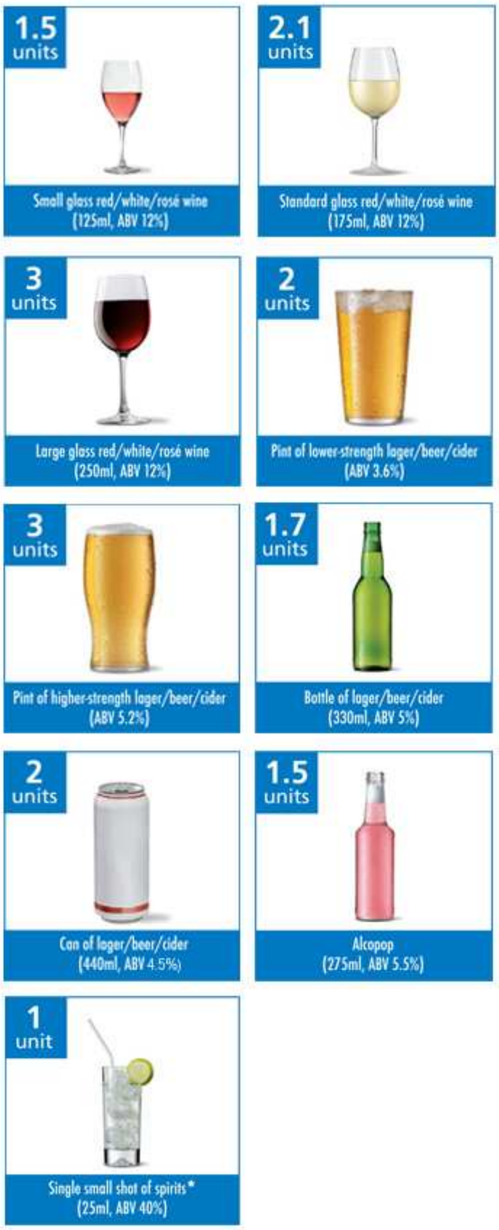

16. Do you have any food allergies?
    ☐ No
    ☐ Yes

    If yes, please tick any allergies that apply below:

    ☐ Tree Nuts (e.g. walnuts, almonds, pine nuts, brazil nuts, and pecans)
    ☐ Peanuts
    ☐ Cow's milk
    ☐ Other milk
    ☐ Eggs
    ☐ Wheat
    ☐ Barley
    ☐ Oats
    ☐ Molluscs
    ☐ Lupin
    ☐ Sesame
    ☐ Sulphites
    ☐ Soy
    ☐ Mustard
    ☐ Celery
    ☐ Fish, shellfish and crustaceans
    ☐ Other, namely……………………………………………………………………..

17. Do you use any dietary supplements? (i.e. vitamin supplements, minerals, fibres or probiotics). Examples of commonly used probiotics include:
    - Actimel drink
    - Activia yogurt
    - Benecol yogurt drink
    - Yakult drink
    - Arla Skyr yogurt drink
    - Creamier bio-live Irish yogurts
    - Probiotics from a local chemist or herbalist (e.g. Acidophilus Capsules)

    ☐ No
    ☐ Yes

    If yes, what products have you used: How often do you use them (e.g. once per day):

    …………………………………………… ……………………………………………

    …………………………………………… ……………………………………………

    …………………………………………… ……………………………………………



Dingena CF, *et al. BMJ Open* 2023; 13:e065388. doi: 10.1136/bmjopen-2022-065388

18. Do you have internet access on a computer, tablet or smartphone at home?
    ☐ Yes
    ☐ No

19. Are you currently participating in any other research studies?
    ☐ Yes
    ☐ No

    If yes, please specify which study and give brief explanation.

    ………………………………………………………………………………………………………………

    ………………………………………………………………………………………………………………

    ………………………………………………………………………………………………………………

    ………………………………………………………………………………………………………………

    ………………………………………………………………………………………………………………

Can we keep this information on file and contact you about future studies? Yes / No

## Suplementary section E: Medical history questionnaire

INFORMED

Individualised patieNt care and treatment FOR MatErnal Diabetes

**Understanding the glycaemic profile of maternal diabetes using continuous glucose monitoring: intensive glucose profiling to inform patient care and treatment**

**Medical History Questionnaire**

1. Do you currently have or have you had history of any of the following diseases?

If you have selected yes for any of the following conditions, please indicate which (if any) you are currently receiving treatment for? Please also state whether the condition limits you in your daily activities.

| | Have you/ are you currently suffering from any of the following? | | Are you receiving treatment for this disease? | | Does this disease limit you in your daily activities? | |
|---|---|---|---|---|---|---|
| **Diseases** | *No* | *Yes* | *No* | *Yes* | *No* | *Yes* |
| Disease of heart/coronary arteries (angina pectoris, heart attack) | | | | | | |
| Hypertension | | | | | | |
| Lung disease | | | | | | |
| Asthma | | | | | | |
| Diabetes | | | | | | |
| Stomach ulcers or other stomach disorders | | | | | | |
| Kidney disease | | | | | | |
| Liver disease | | | | | | |
| Anaemia or any other blood disease | | | | | | |
| Cancer | | | | | | |
| Depression | | | | | | |
| Eating disorder (e.g. anorexia/bulimia) | | | | | | |
| Chronic fatigue syndrome (CFS) | | | | | | |

INFORMED Protocol (version 1.0) IRAS No: 297276 Date: 22-07-2021

| | | | | | | |
|---|---|---|---|---|---|---|
| Stroke | | | | | | |
| Gastrointestinal disease | | | | | | |
| Oesophageal disease | | | | | | |
| Thyroid disease | | | | | | |
| High cholesterol | | | | | | |

| If you are suffering from any other medical problems, please specify below: | *No* | *Yes* | *No* | *Yes* |
|---|---|---|---|---|
| ……………………………………………………………………………………………… ……………………………… | | | | |
| ……………………………………………………………………………………………… ……………………………… | | | | |
| ……………………………………………………………………………………………… ……………………………… | | | | |

2. In the past, have you had any major abdominal surgery (please provide dates)?

Not had any major abdominal surgery in the past

Yes, had major abdominal surgery in the past, namely:

Laparoscopic (or key-hole): appendectomy Cholecystectomy

Open surgery: appendectomy Cholecystectomy

Other abdominal surgery, namely: …………………………………….

Date/s of any surgery:………………………………………………………………………………..

Appendectomy: removal of the appendix

Cholecystectomy: removal of the gall bladder



Dingena CF, *et al. BMJ Open* 2023; 13:e065388. doi: 10.1136/bmjopen-2022-065388

# Suplementary section F: Modified Pregnancy Physical Activity Questionnaire (PPAQ)

INFORMED

Individualised patieNt care and treatment FOR MatErnal Diabetes

**Understanding the glycaemic profile of maternal diabetes using continuous glucose monitoring: intensive glucose profiling to inform patient care and treatment**

**Physical activity questionnaire**

**During this trimester, how much time do you usually spend on:**

1. Preparing meals (cook, set table, wash dishes)

None

Less than ½ hour per day

½ to almost 1 hour per day

1 to almost 2 hour per day

2 to almost 3 hour per day

3 or more hours per day

2. Taking care of an older adult

None

Less than ½ hour per day

½ to almost 1 hour per day

1 to almost 2 hour per day

2 to almost 3 hour per day

3 or more hours per day

3. Sitting and using a computer or writing, <u>not for work</u>

None

Less than ½ hour per day

½ to almost 1 hour per day

1 to almost 2 hour per day

2 to almost 3 hour per day

3 or more hours per day

4. Sitting at work or in class → If the participant does not work or study, skip to question 7.

None

Less than ½ hour per day

½ to almost 1 hour per day

1 to almost 2 hour per day

2 to almost 3 hour per day

3 or more hours per day

5. Standing or walking at work while carrying things (heavier than a 1 gallon milk jug)

None

Less than ½ hour per day

½ to almost 1 hour per day

1 to almost 2 hour per day

2 to almost 3 hour per day

3 or more hours per day

6. Standing or walking at work while not carrying anything

None

Less than ½ hour per day

½ to almost 1 hour per day

1 to almost 2 hour per day

2 to almost 3 hour per day

3 or more hours per day

7. Sitting and reading, talking or on the phone, not for work

None

Less than ½ hour per day

½ to almost 1 hour per day

1 to almost 2 hour per day

2 to almost 3 hour per day

3 or more hours per day

8. Watching TV or video

None

Less than ½ hour per day

½ to almost 1 hour per day

1 to almost 2 hour per day

2 to almost 3 hour per day

3 or more hours per day

9. Light cleaning (make beds, laundry, ironing, putting things away)

None

Less than ½ hour per day

½ to almost 1 hour per day

1 to almost 2 hour per day

2 to almost 3 hour per day

3 or more hours per day

10. Heavier cleaning (vacuum, mop, sweep, wash windows)

None

Less than ½ hour per day

½ to almost 1 hour per day

1 to almost 2 hour per day

2 to almost 3 hour per day

3 or more hours per day

11. Shopping (for food, clothes, or other)

None

Less than ½ hour per day

½ to almost 1 hour per day

1 to almost 2 hour per day

2 to almost 3 hour per day

3 or more hours per day

12. Gardening

None

Less than ½ hour per day

½ to almost 1 hour per day

1 to almost 2 hour per day

2 to almost 3 hour per day

3 or more hours per day

*Please, fill out the next section if you have children. If you do not take care of children, you do not need to complete the this section.*

**During this trimester, taking care of children, how much time do you usually spend on:**

13. Dressing, bathing, feeding children while you are <u>sitting</u>

None

Less than ½ hour per day

½ to almost 1 hour per day

1 to almost 2 hour per day

2 to almost 3 hour per day

3 or more hours per day

14. Dressing, bathing, feeding children while you are <u>standing</u>

None

Less than ½ hour per day

½ to almost 1 hour per day

1 to almost 2 hour per day

2 to almost 3 hour per day

3 or more hours per day

15. Playing with children while you are standing or sitting

None

Less than ½ hour per day

½ to almost 1 hour per day

1 to almost 2 hour per day

2 to almost 3 hour per day

3 or more hours per day

16. Playing with children while you are walking or running

None

Less than ½ hour per day

½ to almost 1 hour per day

1 to almost 2 hour per day

2 to almost 3 hour per day

3 or more hours per day

17. Carrying children

None

Less than ½ hour per day

½ to almost 1 hour per day

1 to almost 2 hour per day

2 to almost 3 hour per day

3 or more hours per day

**During this trimester, how much time do you usually spend on exercising:**

18. Walking to go places (such as to the bus, work, visiting). Not for fun or exercise

None

Less than ½ hour per day

½ to almost 1 hour per day

1 to almost 2 hour per day

2 to almost 3 hour per day

3 or more hours per day

19. Walking for fun or exercise

None

Less than ½ hour per day

½ to almost 1 hour per day

1 to almost 2 hour per day

2 to almost 3 hour per day

3 or more hours per day

20. Jogging

None

Less than ½ hour per day

½ to almost 1 hour per day

1 to almost 2 hour per day

2 to almost 3 hour per day

3 or more hours per day

21. Prenatal exercise class

None

Less than ½ hour per day

½ to almost 1 hour per day

1 to almost 2 hour per day

2 to almost 3 hour per day

3 or more hours per day

22. Doing other things for fun or exercise (such as swimming or dancing)?

None

Less than ½ hour per day

½ to almost 1 hour per day

1 to almost 2 hour per day

2 to almost 3 hour per day

3 or more hours per day

# Suplementary section G: Modified Leeds Sleep Evaluation Questionnaire (LSEQ)

INFORMED

Individualised patieNt care and treatment FOR MatErnal Diabetes

**Understanding the glycaemic profile of maternal diabetes using continuous glucose monitoring: intensive glucose profiling to inform patient care and treatment**

**Sleep quality questionnaire**

How would you describe the way you currently fall asleep in comparison to usual?

1. More difficult than usual -------------- Easier than usual

2. Slower than usual -------------- More quickly than usual

3. I feel less sleepy than usual -------------- More sleepy than usual

How would you describe the quality of your sleep compared to normal sleep?

4. More restless than usual -------------- Calmer than usual

5. With more wakeful periods than usual ------------- With less wakeful periods

How would you describe your awakening in comparison to usual?

6. More difficult than usual -------------- Easier than usual

7. Requires a period of time longer than usual -------------- Shorter than usual

How do you feel when you wake up?

8. Tired -------------- Alert

How do you feel now?

9. Tired -------------- Alert

How would you describe your balance and co-ordination upon awakening?

10. More disrupted than usual -------------- Less disrupted than usual ]

INFORMED Protocol (version 1.0) IRAS No: 297276 Date: 22-07-2021
