# Supplementary material for: Protocol for the INFORMED (Individualised Patient Care and Treatment for Maternal Diabetes) Study: a randomised controlled trial embedded within routine care
Source: BMJ Open. 2023 Feb 27;13(2):e065388. doi: 10.1136/bmjopen-2022-065388 (PMC9972421; doi:10.1136/bmjopen-2022-065388)
Supplement: Author's manuscript [file bmjopen-2022-065388.draft_revisions.pdf]

BMJ Open

BMJ Open is committed to open peer review. As part of this commitment we make the peer review history of every article we publish publicly available.

When an article is published we post the peer reviewers' comments and the authors' responses online. We also post the versions of the paper that were used during peer review. These are the versions that the peer review comments apply to.

The versions of the paper that follow are the versions that were submitted during the peer review process. They are not the versions of record or the final published versions. They should not be cited or distributed as the published version of this manuscript.

BMJ Open is an open access journal and the full, final, typeset and author-corrected version of record of the manuscript is available on our site with no access controls, subscription charges or pay-per-view fees (http://bmjopen.bmj.com).

If you have any questions on BMJ Open's open peer review process please email info.bmjopen@bmj.com

BMJ Open

Protocol for the INFORMED (Individualised Patient Care and Treatment for Maternal Diabetes): A randomised controlled trial embedded within routine care

Journal:	*BMJ Open*
Manuscript ID	bmjopen-2022-065388
Article Type:	Protocol
Date Submitted by the Author:	07-Jun-2022
Complete List of Authors:	Dingena, Cassy; University of Leeds, Food Science and Nutrition<br>Mahendra, Anvesha; University of Leeds, Food Science and Nutrition<br>Holmes, Melvin; University of Leeds, Food Science and Nutrition<br>Clement, Naomi S; University of Leeds, Medicine<br>Scott, Eleanor; University of Leeds, Division of Clinical and Population Sciences, Leeds Institute of Cardiovascular and Metabolic Medicine<br>Zulyniak, Michael; University of Leeds
Keywords:	NUTRITION & DIETETICS, Diabetes in pregnancy < DIABETES & ENDOCRINOLOGY, General diabetes < DIABETES & ENDOCRINOLOGY

SCHOLARONE™
Manuscripts

1

TITLE: Protocol for the INFORMED (Individualised Patient Care and Treatment for Maternal Diabetes): A randomised controlled trial embedded within routine care

AUTHORS: Cassy Dingena[1], Anvesha Mahendra[1], Melvin Holmes[1], Naomi S Clement[2], Eleanor Scott [3] and Michael A. Zulyniak[1*]

AUTHOR AFFILIATIONS:

[1] Nutritional Epidemiology Group, School of Food Science and Nutrition, University of Leeds, Leeds, UK.

[2] School of Medicine, University of Leeds, UK.

[3] Division of Clinical and Population Sciences, Leeds Institute of Cardiovascular and Metabolic Medicine, University of Leeds, Leeds, UK.

* CORRESPONDING AUTHOR: Michael A. Zulyniak, Nutritional Epidemiology Group, School of Food Science and Nutrition, University of Leeds, Leeds, LS2 9JT. Email: m.a.zulyniak@leeds.ac.uk. Ph. +44(0)1133430685

WORD COUNT: 3650

KEYWORDS: pregnancy, CGM, glucose, dysglycemia, RCT, nutrition

For peer review only

ABSTRACT

Introduction: Diabetes in pregnancy presents a unique physiological challenge to manage glycaemia, while maintaining adequate nourishment for the growing foetus. Women with diabetes who become pregnant are at greater risk of adverse maternal and newborn outcomes, compared to non-diabetic women. Evidence suggests that control of (post-prandial) glycaemia is key to manage maternal and offspring health but it is not yet clear what aspects of maternal and offspring health are mediated (i) directly by dysglycemia or (ii) shifts in overall metabolism, and how diet and lifestyle moderate these shifts across the full duration of pregnancy. To investigate these, a double-blind, cross-over randomised clinical trial has been embedded within routine clinical care.

Methods and Analysis: Seventy-six pregnant women in their 1st trimester with T1D/T2D (with or without medication) attending their routine antenatal appointments at NHS Leeds Teaching Hospitals will be recruited. Following informed consent, data on women's health, glycaemia, pregnancy, and delivery will be shared by the NHS with researchers. At each visit in the 1st (10-12 weeks), 2nd (18-20 weeks), and 3rd (28-34 weeks) trimester participants will be asked for consent to: (i) lifestyle and diet questionnaires, (ii) blood for research purposes, and (iii) analysis of urine collected at clinical visits. Additionally, participants will be asked to consume two blinded meals in duplicate in 2nd and 3rd trimester. Glycemia will be assessed by continuous glucose monitoring as part of routine care. Primary outcomes include (i) effect of experimental meals on glycaemia, and (ii) association between dysglycemia and maternal and newborn health. Secondary outcomes will investigate the association between maternal metabolic profiles in early pregnancy with dysglycemia in later pregnancy.

Ethics And Dissemination: The Leeds East Research Ethics Committee and NHS approved the study. Results will be published in peer-reviewed journals and disseminated to participants and the wider public.

ISRCTN: 57579163 (Protocol CPMS 50813)

REC: 21/NE/0196.

For peer review only

3

INTRODUCTION

Background & Scope

Pregnancy naturally induces a state of mild insulin resistance (IR) to shuttle more nutrients to the growing baby; however, in women with diabetes in pregnancy (DIP), excessive insulin resistance and persistent hyperglycaemia increases the risk of adverse pregnancy outcomes[1-4]. Globally, the prevalence of DIP is on the rise, affecting ~17% of all pregnancies[4 5]. Compared to women without diabetes, women with DIP are at elevated risk of pre-eclampsia, preterm delivery, and mortality, while their offspring are at increased risk of unhealthy weight (<2.5 kgs or >4.5 kgs.), dysglycemia, injuries at birth, and higher risk of type 2 diabetes mellitus (T2DM) and cardiovascular disease (CVD) in later life[1 5 6].

Post-prandial glycaemic control is important for healthy pregnancy outcomes[1 6]. Evidence supports a healthy diet and lifestyle – that includes wholegrains, fruits and vegetables, and regular physical activity – as the cornerstone for managing DIP, which is effective in 70-85% of women with DIP[7-9]. NICE UK guidelines primarily focus on improving carbohydrate quality by including more low glycaemic index (GI) foods as part of a balanced diet including wholegrains, fruits and vegetables to manage glycaemia during pregnancy [7]. Although low GI diets do support the management of mean glucose levels, their effect on reducing episodes of hypo- and hyperglycaemia and ability to reduce maternal and offspring risk of complications is not clearly established [(10)]. However, some women find it challenging to consistently follow a balanced diet, due to barriers such as availability, accessibility and affordability of healthy foods, lack of time and cooking skills [(7 & 9)]; therefore, a cost-effective nutritious meal replacement may be useful for supporting healthy eating habits. Additionally, emerging evidence in preclinical and humans studies, suggest that the amount of maternal protein intake can improve management of dysglycemia in DIP [(11)].

Continuous glucose monitors (CGMs) are becoming routinely used in the UK National Health Service in perinatal clinical settings for women with DIP[1]. The unobtrusive patches record an individual's glucose every 5 mins for up to 14 days and offer quantitative information to identify interstitial glucose deviations over a 24-hr period. By measuring glucose continuously over hours and days, a more complete representation of dysglycemia can be modelled and offer novel insight regarding the parameters that drive and associate with dysglycaemia, and their impact on maternal and offspring health[10].

For peer review only

Previous studies[11 12] have uncovered new associations and identified novel points of interest for managing dysglycemia during pregnancy and mediating health risks. However, our ability to inform new strategies to manage these new areas of concern are limited by our understanding of the contribution of biological, lifestyle, and environment exposures on dysglycemia in early, mid, and late pregnancy and their moderating effect on maternal and offspring health. To address this gap in current understanding, this study aims to investigate the effect of dietary mediators on (i) 7-day glucose variability in early, mid, and late pregnancy; (ii) and their association with maternal and offspring health during pregnancy and at delivery; and (iii) the moderating effect of biology, ethnicity, metabolism, and lifestyle factors on glycemia throughout pregnancy affected by Type 1 or Type 2 Diabetes.

Aim And Objectives

Our aim is to investigate post-prandial CGM glucose profiles throughout the course of the pregnancy and how they are associated with personal (lifestyle) characteristics and physiological parameters. Our primary research objective is to assess the role of diet as a mediator of dysglycemia over the course of pregnancy. Secondary research objectives will determine the personal characteristics that drive differences in dysglycemia before and after a meal.

METHODOLOGY AND ANALYSIS

Participants

Women with type 1 and 2 diabetes mellitus (T1DM and T2DM) during pregnancy in their 1st trimester will be recruited from the DIP antenatal clinics at Leeds Teaching Hospitals NHS Trust (LTHT). Women will be approached by their direct clinical care team and given a study information flyer and invited to contact the research team (phone or email) if they are interested to participate or if they wish to discuss the study in more detail. Women expressing interest at the end of the initial meeting will be emailed a participant information sheet and a web link to a secure electronic informed consent. Once a secure electronic signature is provided, the participant's eligibility will be assessed according to study inclusion and exclusion criteria.

Sample size using power calculation

There is no current evidence regarding the effect of diet composition and glycaemic load on metrics of continuous glucose monitoring (CGM) in pregnant women with diabetes throughout

For peer review only

the duration of pregnancy. However, evidence from Fabricatore et al (2011)[13] demonstrated a significant association ($p<0.05$) between self-reported glycemic index(GI) and measures of CGM (including AUC, mean glucose, and % time hyperglycemic) in a clinical trial of 21 women and 5 men with type-2 diabetes. Assuming similar effect sizes between GI (per unit) and AUC glucose (β=0.36 mg/dl/min; R^2=0.38), mean glucose (β=0.02 mol/L; R^2=0.38), and time spent > 10 mol /L blood glucose (β=0.41%; R^2=0.36), we will have sufficient power (1-β = 0.90) to detect a significant pairwise effect of GI on these parameters with 63 participants. Another study by Law et al (2019) suggested that this will provide sufficient power (1- β = 0.90) to compare subgroups of participants (stratified by BMI, age, type of diabetes) and test for significant differences (of a minimum effect size) in mean glucose (± 0.5 mmol/L), AUC glucose (± 61 mmol/L/min), and % time hyperglycaemic (± 3.7 %). Finally, given the comparable proportions of women reported to be of white European vs non-white European descent (57% vs 43%) or diet vs diet+ medication (46% vs 54%) we also anticipate having adequate power to compare these confounders of glycaemic response. To account for attrition, we will increase or recruitment target by 20% above our calculated, suggesting a target sample size of 76 recruited women. We have allocated ~6 months to recruit 76 women (at 10-12 weeks gestation) and ~15 months for study completion (i.e., final delivery).

Study participation inclusion and exclusion criteria

All pregnant women over the age of 18, with pre-existing T1DM or T2DM, in their 1st trimester and a singleton pregnancy, will be considered for the study. Exclusion criteria include: (i) inability to understand English sufficiently to read the participant information sheet (PIS) and provide consent (**Supplementary Material, A-C)**; (ii) mental and/or psychological disorders that undermine informed consent; (iii) cancer, digestive tract disorders; (iv) lack of internet access on a computer or tablet at home. Detailed exclusion criteria in **Table 1** (Study screening questionnaires in **Supplementary Material, D and E**).

Table 1: Study participant inclusion and exclusion criteria

INCLUSION CRITERIA	EXCLUSION CRITERIA
Women aged 18-45 years	Women under 18 or above 45 years of age
Singleton pregnancy	Multiple pregnancy
Women in the first trimester of pregnancy	Foetal congenital abnormality
Previously diagnosed with type 1 or type 2 diabetes mellitus.	No diagnosis of diabetes
	Diagnosis of gestational diabetes

For peer review only - http://bmjopen.bmj.com/site/about/guidelines.xhtml

	Significant co-existent medical condition (e.g. overt diabetes complications, cancer, gut mobility or digestion disorder)
	Significant psychological (e.g. anorexia, Bulimia) and/or mental disorders which undermines informed consent
	Dietary allergies or intolerance for the experimental meals
	Lack of internet access on a computer or tablet at home
	Unable to understand written English and provide informed consent

Data collection stages and procedures

The study will proceed in three stages at the St James's hospital (LTHT) with the dietary intervention and interviews conducted remotely (e.g., participant's home) (**Fig 1**). All pregnant women with pre-existing T1DM or T2DM are scheduled for regular NHS clinical visits every two weeks throughout the pregnancy. Each woman has an assigned diabetes midwife who caseloads her pregnancy and liaises with the rest of the clinical care team. Due to COVID-19 and intermittent lockdowns, pregnant women only attend face-to-face meetings at the clinic when due for a scan (*a dating scan* at 10-12 weeks, an *anomaly scan* at 18-20 weeks and *growth scans* at 26-28, 32-34, and 36 weeks of pregnancy) unless deviated due to complications or early delivery. All women with T1D and T2D are currently offered CGM as part of their clinical care. The CGM data is automatically uploaded to a secure, remote clinical database, where it can be securely accessed and downloaded by the clinical team and authorised researchers.

The study will require patient consent (i) permitting secure access to routinely collected clinical details regarding maternal and offspring health at each clinical visit and delivery (i.e., height, weight, blood pressure, HbA1c, lipids), continuous glucose monitoring (CGM) data, and delivery outcomes with approved study researchers; (ii) permitting researchers to use the residual urine from routine clinically collected samples for metabolite analysis; (iii) to conduct online and interview questionnaires to assess diet and lifestyle during each trimester at ~10-12 weeks, ~18-20 weeks, and ~28-34 weeks; and (iv) a 10ml blood sample to be taken with routine clinical bloods at ~10-12 weeks, ~18-20 weeks, and ~28-34 week visits for subsequent metabolomic and genetic analysis. Each participant will be contacted three times for a phone or video chat (participant preference) for ≤ 30 minutes each within 2 weeks of each clinical appointment.

For peer review only

Patient and Public Involvement: Patients or the public have not been involved in the design of this pilot and feasibility study. However, upon completion of the study, participants will be invited to provide insight and comments regarding the study itself, the burden enrolment and intervention, and identifying other research priorities relevant to the health condition that the researchers can integrate into future studies. They will also be asked if they consent to follow-up discussions and are keen to be updated on study results and publication material. These points will be invaluable for guiding future work in this area.

First call 10-12 weeks of pregnancy: A member of the research team will provide details of the study with the participant and answer any questions. The participants will be instructed to record their dietary intake for three days, including two weekdays and one weekend, using the myFood24 app. MyFood24 is a validated online food diary system created to analyse nutritional intake, which has previously been used in pregnancies complicated by diabetes[14]. Details on participant's recent physical activity levels, sleep quality or patterns and habitual mealtimes will be recorded by interview. The dietary meal intervention in the study will also be discussed.

Second call between 18-20 weeks of pregnancy: Participant's compliance in the study and details on physical activity, sleep and habitual mealtimes will be re-recorded. A reminder and summary of when and how to use the myfood24 app will be given. The participant will also be instructed about how and when their study intervention's first set of experimental meals should be consumed.

Third call between 28-34 weeks of pregnancy: Participant's compliance and information transfer as in the second call will be repeated. The participant will also be instructed about how and when their study intervention's second set of experimental meals should be consumed. Subsequently, the participant will be thanked for their participation in the study and not contacted again after this point.

Nested cross-over dietary meal intervention within the study

Shortly after their ~18-20 week and ~28-34-week clinical visit, participants will be asked to consume standardised breakfast meal replacements in their own home, at breakfast time for 4 days. These will be two different experimental meals (A and B; matched for 400 kcal and 13 g of fat) consumed under free-living conditions. The experimental meals will appear and taste similar but differ in protein quantity which will alter how quickly the glucose in each meal is absorbed into the blood[15]. One experimental meal will have 20g of vegan and gluten-free

For peer review only

protein powder added, which slow gastric emptying and glucose absorption into the blood to rate that is comparable to commonly consumed whole grain breakfast cereals (e.g., steel-cut or rolled oats; GI≈40). The other experimental meal will have no added protein. The experimental meals (labelled A or B) with a drink shaker in a box by the research team in a COVID-safe lab. Each participant will be assigned to one of six random orders to consume the experimental breakfast meals (AABB, ABAB, BBAA, BABA, ABBA, BAAB) over 4 days using an online randomiser (https://www.random.org/integer-sets/). The participant will only need to pour the powder into the shaker, add cold water to the line marked on the cup (500 mL), and consume within 5 minutes.

The powder is a nutritionally complete meal replacement in drink form, every meal contains a balance of protein, carbohydrates, essential fats, fibre, plus all 26 essential vitamins and minerals, and phytonutrients. Additionally, the product is low in sugar, lactose-free, contains no nuts or palm oil, and has a long shelf life. The powder is commercially available and is produced in facilities that meet highest quality standards. This product was chosen to minimise time-burden for participants and it is free from many commonly avoided food items (i.e., lactose, nuts, gluten, and meat).

Participants will be asked to avoid consuming other foods and drinks (aside from water) for two hours, after which they may consume food as usual. However, they are freely permitted to measure their own blood glucose levels at any point and will be advised to manage any hyperglycaemic or hypoglycaemic events, even if this means eating or drinking within 2 hours of the meal. We will ask that they inform the research team of any events by email as soon as possible and these will be discussed any follow up meetings.

Data management

The research team will assign unique random screening IDs at the recruitment phase. The study ID will be used to pseudonymise (using personal participant identification numbers; PID) and harmonise the data shared by the NHS clinical database (clinical records) with the data collected by the research team at the University of Leeds (i.e., questionnaires, metabolite, and genetic data). Only the clinical team and authorised members of the research team will be able to link the study ID to the participant. All personally identifiable information will be stored in a password protected and encrypted database in a secure area.

For peer review only - http://bmjopen.bmj.com/site/about/guidelines.xhtml

Clinical data: Standard care to measure and collect maternal anthropometric, glycaemic, medication, lipid levels, and blood pressure information during routine hospital visits and during and after labour. Offspring anthropometry measures are taken by the direct healthcare team, which is part of routine care. The women will be asked to give consent for the research team to access their clinical records to obtain this data.

CGM: Standard clinical care in T1D and T2D pregnancies, women will be asked to give consent for the research team to access their CGM data.

Urine samples: Standard clinical care, we will ask for up to 2.5 mL of any urine not required for clinical analysis to be saved for research use. These samples will be stored for subsequent metabolic analysis. All samples will be stored at the University of Leeds in designated Human Tissue Act approved and compliant facilities.

Blood samples: Standard clinical care requires blood samples for analysis. At the time of routine collection at each clinical visit, an additional 10ml blood will be collected for this study. These blood samples will be stored for subsequent metabolic and genomic analysis (relevant to nutrition/diabetes/pregnancy and fetal growth). All samples will be stored at the University of Leeds in designated and secure facilities.

Lifestyle questionnaires: At three time points across pregnancy (~10-12, ~18-20, and ~28-34 weeks gestation), a designated member of the research team will call (phone or video) the participant to complete the questionnaires on physical activity, sleep quality/patterns, and habitual mealtime habits.

Dietary records: At two timepoints across pregnancy (~18-20 and ~28-32 weeks of gestation), each participant will be asked to record their diet for 3 days (2 week days, 1 weekend day) using an online food frequency questionnaire (myFood24).

Outcomes of interest: The primary outcome of interest is CGM glucose data. Secondary outcomes of interest are associations between metrics of dysglycemia during pregnancy with maternal outcomes (e.g. gestational weight gain, pre-eclampsia, hypertension, mode of birth, birth trauma, pre-term delivery, and metabolism), and infant outcomes (e.g. birth weight, height, pre-term delivery, mortality, birth trauma, hypoglycaemia, congenital malformation, head and abdominal circumference, perinatal morbidity), and the moderating effects of genetics, metabolism, and diet and lifestyle. All secondary analyses are considered exploratory.

For peer review only

Statistical analysis considerations

A panel of standard CGM metrics (such as mean glucose, AUC, coefficient of variation and time in/above/below target range) will be calculated as measurements of glycaemic control and presented as mean±SD. The association between habitual diet (food diary using Myfood24) and glycemic control at each trimester), will be performed using three distinct dietary metrics to differentiate dietary drivers:

(1) *Overall diet quality*: The association between diet quality and dysglycemia will be assessed using an overall diet quality score[16]. This scoring method has been modified and used previously to assess maternal diet quality in a multi-ethnic prospective birth cohort[17]. The modified Alternative Healthy Eating Index (mAHEI) score is calculated using the following method; an individual will receive 10 points for each of the following food categories when they consume above or below a threshold of: ≥5 servings vegetables, ≥4 servings fruit, ≥1 serving nuts or soy proteins, ≥3 servings whole grains, a ratio of ≥4 servings fish to 1 serving meat and eggs, and ≤0.5 servings of less-healthy foods (i.e., fried foods and processed meats). Intermediate intakes are scored proportionally between 0 and 10. The maximum mAHEI score is 60; the higher the score the more healthful the participant's diet.

(2) *Macronutrient*: Daily macronutrient consumption (total carbohydrates, proteins, and fats) and markers of GI quality (fibre, sugars) will be adjusted for energy and regressed against CGM measures of dysglycemia. Doing so, will allow for the contribution of individual macronutrients on glycemic measures to be evaluated.

(3) *Cardinal Foods*: Partial-least Squares (PLS) will be used to identify foods that are more commonly observed in participants with favourable or unfavourable glycemic control (identified above/below median for glycemic measures). These foods will then be investigated for their association with measures of dysglycemia using a regression model.

Analyses will be adjusted for covariates (e.g., maternal age, ethnicity, parity, BMI, gestational age) and potential lifestyle mediators of glycemic response (e.g. physical activity and sleep quality). Changes to the effect sizes and p-values of glycemic measures will be monitored when lifestyle parameters are added to identify mediators of glycemic response. The effect of diet composition on glycemic control will be investigated using t-tests and regression models. T-tests will be used to confirm our hypothesis that the meals that differ in rate of glucose absorption will elicit different glycemic responses but that these associations are moderated by biological and lifestyle factors — e.g., diet (i.e., quality, macronutrients, specific foods) physical activity levels, BMI, sleep, metabolism. Changes to the effect sizes or level of

For peer review only

significance of glycemic measure would suggest a meaningful effect of the moderator on glycaemia. Statistical significance will be set at $p<0.05$. Statistical analysis will be conducted in R studio and SPSS.

Quality control: All participants will receive standard clinical care as per NICE guidance, which will minimise researcher bias. The primary outcome measures are based on laboratory measurements and predetermined cut-off values, which the researchers will not be able to influence. We do not foresee significant researcher bias in collecting antenatal and perinatal outcome data because these will be obtained by clinical staff who are independent of the study outcome and from the participants' medical records. We do not foresee any significant researcher bias in collecting lifestyle records, as standardised and validated questionnaires will be used to obtain this information. Furthermore, all participants will be asked to consume the two standard meals but the order of their consumption will be randomised. We do not foresee any conflict of interests. The data collected will not be used for informing clinical care decisions of specific cases, all the women will continue with their usual clinical care pathway for the duration of their pregnancy, and all women will be free to terminate their participation in the study at any time with no effect on their quality of care.

ETHICS & DISEMMINATION

Ethics approval: This study has been reviewed and approved by the Leeds East Research Ethics Committee at the University of Leeds (21/NE/0196).

Dissemination: There is no formal interim analysis planned except the ongoing evaluation of the recruitment numbers. Results will be disseminated in peer reviewed scientific journals, conference presentations, and publication on (social) media and in newsletters, to inform the participants and wider public.

DISCUSSION

The INFORMED clinical trial is double-blinded cross-over randomised clinical trial to evaluate the safety and efficacy of experimental meals that differ in rate of glucose absorption on glucose variability during pregnancy (using CGM) in women with pre-existing type-1 or type-2 diabetes throughout the duration of pregnancy. Additionally, the analysis of 'omics' technologies, particularly repeated metabolomics-based analyses, as predictors of

For peer review only

dysglycaemia management, may provide insights into novel precision therapies for women with DIP[18]. The identification of dietary mediators of glucose variability will aid in the development of more efficacious and appropriate strategies to control glucose levels and minimise maternal and offspring risks in women with DIP[11 12 18].

Strengths & limitations:

The trial is embedded within standard clinical care and uses routine data and biological samples collected by health services to minimise (i) participant burden, and (ii) non-essential participant contact, which reduces risk of bias and will permit the study to continue even with the re-introduction of COVID-based restrictions. Analyses of response to meals alongside repeated measures of blood and urine metabolite profiles, will offer insights into distinct shifts in metabolism during pregnancy in type-1 and type-2 diabetes, and their association with maternal and newborn health at delivery.

INFORMED is being conducted with the National Health Service (NHS) of the UK; therefore, its results may not be directly generalizable to other nations or government health services.

AUTHOR CONTRIBUTIONS: **Cassy Dingena**: Conception, original draft preparation, writing - review & editing, approved the final manuscript. **Anvesha Mahendra**: Original draft preparation, writing - review & editing, approved the final manuscript. **Melvin Holmes**: Supervision, Writing - review & editing, approved the final manuscript. **Naomi S Clement**: Writing - review & editing, approved the final manuscript. **Eleanor Scott**: Conception, supervision, writing - review & editing, approved the final manuscript. **Michael Zulyniak**: Conception, supervision, Original draft preparation, writing - review & editing, approved the final manuscript.

ACKNOWLEDGEMENTS: myFood24 was developed through Medical Research Council funding, grant G110235. myfood24 is now being supported by spinout company Dietary Assessment Ltd. Requests to use myfood24 should be made to enquiries@myfood24.org.

FUNDING STATEMENT: This work was supported by Wellcome Trust UK (MAZ; 217446/Z/19/Z) and the University of Leeds (CD; N/A).

COMPETING INTEREST STATEMENT: No competing interest.

For peer review only

13

REFERENCES:

1. Macintosh MCM, Fleming KM, Bailey JA, et al. Perinatal mortality and congenital anomalies in babies of women with type 1 or type 2 diabetes in England, Wales, and Northern Ireland: population based study. *Bmj* 2006;333(7560):177.
2. Murphy HR, Howgate C, O'Keefe J, et al. Characteristics and outcomes of pregnant women with type 1 or type 2 diabetes: a 5-year national population-based cohort study. *The lancet Diabetes & endocrinology* 2021;9(3):153-64.
3. Symonds ME, Ramsay MM. Maternal-fetal nutrition during pregnancy and lactation: Cambridge University Press 2010.
4. Lapolla A, Metzger BE. Gestational Diabetes: A Decade After the HAPO Study: Karger Medical and Scientific Publishers 2019.
5. Federation ID. International Diabetes Federation. IDF Diabetes Atlas, 9th edn. Brussels, Belgium: 2019, 2019.
6. Tennant PWG, Glinianaia SV, Bilous RW, et al. Pre-existing diabetes, maternal glycated haemoglobin, and the risks of fetal and infant death: a population-based study. *Diabetologia* 2014;57(2):285-94.
7. National Institute for Clinical E. Diabetes in pregnancy: management from preconception to the postnatal period. *NICE Guidelines [NG3] Published February* 2015
8. Smith R, Ridout A, Livingstone A, et al. Motivational interviewing to increase physical activity in women with gestational diabetes. *British Journal of Midwifery* 2021;29(10):550-56.
9. American Diabetes A. Management of diabetes in pregnancy. *Obstetrical & Gynecological Survey* 2017;72(5):264-66.
10. Yu Q, Aris IM, Tan KH, et al. Application and utility of continuous glucose monitoring in pregnancy: a systematic review. *Frontiers in Endocrinology* 2019:697.
11. Perichart-Perera O, Balas-Nakash M, Rodríguez-Cano A, et al. Low glycemic index carbohydrates versus all types of carbohydrates for treating diabetes in pregnancy: a randomized clinical trial to evaluate the effect of glycemic control. *International journal of endocrinology* 2012;2012
12. Moses RG, Luebcke M, Davis WS, et al. Effect of a low-glycemic-index diet during pregnancy on obstetric outcomes. *The American Journal of Clinical Nutrition* 2006;84(4):807-12.
13. Fabricatore AN, Ebbeling CB, Wadden TA, et al. Continuous glucose monitoring to assess the ecologic validity of dietary glycemic index and glycemic load. *The American journal of clinical nutrition* 2011;94(6):1519-24.
14. Morris MA, Hutchinson J, Gianfrancesco C, et al. Relationship of the frequency, distribution, and content of meals/snacks to glycaemic control in gestational diabetes: the myfood24 GDM pilot study. *Nutrients* 2019;12(1):3.
15. Meng H, Matthan NR, Ausman LM, et al. Effect of macronutrients and fiber on postprandial glycemic responses and meal glycemic index and glycemic load value determinations. *The American journal of clinical nutrition* 2017;105(4):842-53.
16. Dehghan M, del Cerro S, Zhang X, et al. Validation of a semi-quantitative Food Frequency Questionnaire for Argentinean adults. *PLoS One* 2012;7(5):e37958.

For peer review only

17. NutriGen Alliance I, de Souza RJ, Zulyniak MA, et al. Harmonization of food-frequency questionnaires and dietary pattern analysis in 4 ethnically diverse birth cohorts. *The Journal of nutrition* 2016;146(11):2343-50.
18. Schaefer-Graf U, Napoli A, Nolan CJ. Diabetes in pregnancy: a new decade of challenges ahead. *Diabetologia* 2018;61(5):1012-21.

For peer review only

FIGURE LEGENDS

Figure 1. Study Flowchart. Participant will be monitored and in contact with the clicnial and research team through the sutdy, starting at 10-12 weeks gestation. At each clinical visit, routine data will be collected from each particpant as stadard of care (e.g., anthropometrics, blood samples and CGM). This informaiton will be supplemented with a lifestyle information (e.g., diet and sleep) collected directly from the particiapnt via electronic and internet questionaires. The maternal and offspring data available for analysis at each timepoint is listed below the timeline. The interventions will be delivered at two timepoints (18-20 weeks and 34-36 weeks). CGM: continuous glucose monitoring

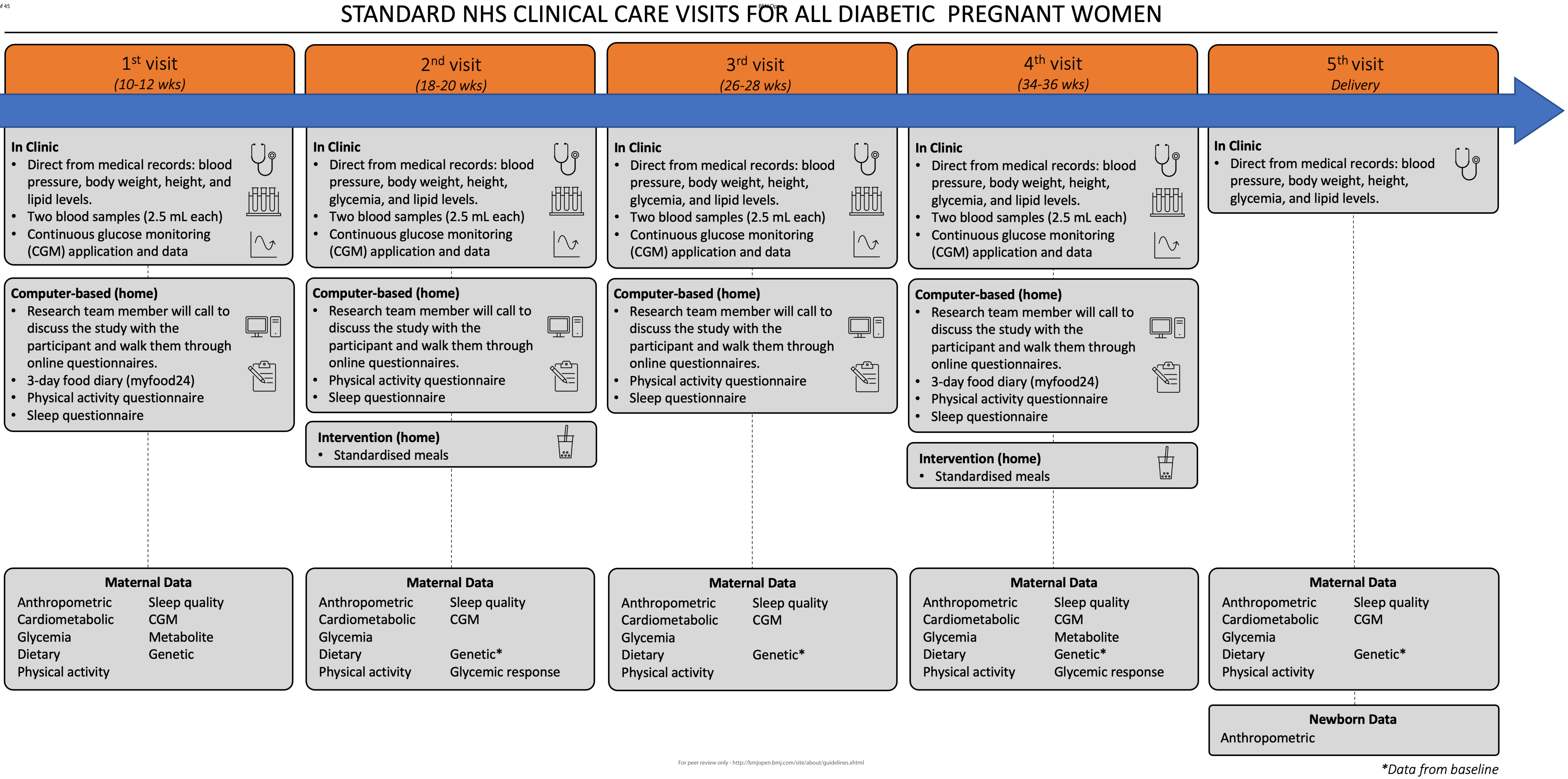
STANDARD NHS CLINICAL CARE VISITS FOR ALL DIABETIC PREGNANT WOMEN
1st visit (10-12 wks)
2nd visit (18-20 wks)
3rd visit (26-28 wks)
4th visit (34-36 wks)
5th visit Delivery
In Clinic
• Direct from medical records: blood pressure, body weight, height, and lipid levels.
• Two blood samples (2.5 mL each)
• Continuous glucose monitoring (CGM) application and data
Computer-based (home)
• Research team member will call to discuss the study with the participant and walk them through online questionnaires.
• 3-day food diary (myfood24)
• Physical activity questionnaire
• Sleep questionnaire
Maternal Data
Anthropometric
Cardiometabolic
Glycemia
Dietary
Physical activity
Sleep quality
CGM
Metabolite
Genetic
In Clinic
• Direct from medical records: blood pressure, body weight, height, glycemia, and lipid levels.
• Two blood samples (2.5 mL each)
• Continuous glucose monitoring (CGM) application and data
Computer-based (home)
• Research team member will call to discuss the study with the participant and walk them through online questionnaires.
• Physical activity questionnaire
• Sleep questionnaire
Intervention (home)
• Standardised meals
Maternal Data
Anthropometric
Cardiometabolic
Glycemia
Dietary
Physical activity
Sleep quality
CGM
Genetic*
Glycemic response
In Clinic
• Direct from medical records: blood pressure, body weight, height, glycemia, and lipid levels.
• Two blood samples (2.5 mL each)
• Continuous glucose monitoring (CGM) application and data
Computer-based (home)
• Research team member will call to discuss the study with the participant and walk them through online questionnaires.
• Physical activity questionnaire
• Sleep questionnaire
Maternal Data
Anthropometric
Cardiometabolic
Glycemia
Dietary
Physical activity
Sleep quality
CGM
Genetic*
In Clinic
• Direct from medical records: blood pressure, body weight, height, glycemia, and lipid levels.
• Two blood samples (2.5 mL each)
• Continuous glucose monitoring (CGM) application and data
Computer-based (home)
• Research team member will call to discuss the study with the participant and walk them through online questionnaires.
• 3-day food diary (myfood24)
• Physical activity questionnaire
• Sleep questionnaire
Intervention (home)
• Standardised meals
Maternal Data
Anthropometric
Cardiometabolic
Glycemia
Dietary
Physical activity
Sleep quality
CGM
Metabolite
Genetic*
Glycemic response
In Clinic
• Direct from medical records: blood pressure, body weight, height, glycemia, and lipid levels.
Maternal Data
Anthropometric
Cardiometabolic
Glycemia
Dietary
Physical activity
Sleep quality
CGM
Genetic*
Newborn Data
Anthropometric
*Data from baseline

UNIVERSITY OF LEEDS

The Leeds Teaching Hospitals NHS Trust

INFORMED

Individualised patieNt care and treatment FOR MatErnal Diabetes

SUPPLEMENTARY MATERIALS

Supplementary section: A

INFORMED

INDIVIDUALISED PATIENT CARE AND TREATMENT FOR MATERNAL DIABETES

UNIVERSITY OF LEEDS

NHS The Leeds Teaching Hospitals NHS Trust

THE MAIN ISSUE?

A mother's blood **glucose changes** after mealtimes and throughout the day, affected by her **personal characteristics**, daily lifestyle and the pregnancy itself. **Too much or uncontrolled glucose** in your blood during pregnancy can lead to a large baby and can cause problems during pregnancy and labour. Also, babies exposed to higher glucose levels are more likely to become **obese** and get **Type 2 diabetes** when they grow up.

WHAT WILL WE INVESTIGATE?

Recent studies have shown that **other factors beyond the characteristics of food** play an important role in how glucose is absorbed after a meal during pregnancy. These factors include your **personal charactristics** such as age, ethnicity and BMI and genetics.

Using **continuous glucose monitoring** (CGM), which measures **glucose levels every few minutes**, we will investigate:

1) How **diet quality effects glucose control** in type 1 and 2 diabetes pregnancy?
2) Which **personal characteristics** are most strongly related?
3) How does **glucose control** evolve during pregnancy?

INTERESTED?

If you have **type 1 or type 2 diabetes** and are **within the first 12 weeks of pregnancy** and interested in taking part, please **contact us** for more information.

+31627072821

fscd@leeds.ac.uk

WHAT WE WILL ASK FROM YOU?

During your routine care, medical details are recorded and you will wear a CGM device. We ask for your permission to safely access and assess this data.

Furthermore, we ask you to complete short questionnaires on diet, physical activity and sleep at three occasions during your pregnancy (after each routine care visit). These questionnaires will be partly online and via phone calls.

To gain more insight in mealtime glucose responses, we ask you to consume standardised breakfast meals on two separate occasions (optional), no addtional clinical visits needed. These meals will be delivered at your home.

HOW LONG DOES THE STUDY LAST?

We will collect data throughout the pregnancy, inluding birth outcomes.

WHAT WILL THIS TELL US?

We think that all the information we gather will help to develop new ways in which women can reduce their risk of having uncontrolld glucose, reduce still births, pregnancy complications and improve the long term health of their children.

INFORMED Infographic (version 1.0) IRAS No: 297276 Date: 02-07-2021

For peer review only - http://bmjopen.bmj.com/site/about/guidelines.xhtml

UNIVERSITY OF LEEDS

The Leeds Teaching Hospitals NHS Trust

Supplementary section B: Participant Information Sheet

INFORMED

Individualised patieNt care and treatment FOR MatErnal Diabetes

Understanding the glycaemic profile of maternal diabetes using continuous glucose monitoring: intensive glucose profiling to inform patient care and treatment

Participant Information Sheet

Thank you for considering your participation in our study called INFORMED, which is part of a PhD research project at the School of Food Science and Nutrition. We - as research team - would like to provide you with details about the study, what your role will involve, and other key information before you decide.

Please ask us (*contact details at the end of the handout*) if there is anything that is not clear or you would like more information. Take time to decide whether or not you wish to take part.

Study information

What is the purpose of the study?
During pregnancy, a mother's blood glucose level changes constantly across 24-hour period, and is affected by her physical characteristics, lifestyle, and the pregnancy itself. While many factors affect the way babies grow in the womb, one of the easiest to measure and modify, is the amount of glucose that they get from their mother. Uncontrolled or too much glucose in their mother's blood during pregnancy, usually leads to a large baby and can increase the chance of problems during pregnancy, labour, and immediately after birth for both mother and child. Being born too small can also be problematic and has been linked to increases the chances of obesity and type 2 diabetes.

Glucose levels of the mother rise after meal consumption and, if uncontrolled, can contribute to some of these health concerns. While the type of food being eaten is vital, recent studies have shown that other factors (such as age, ethnicity, activity levels, and sleep duration) also play a part. However, despite knowing these factors, we currently do not know how to modify a meal to match a mother's characteristics and how a mother's diet effects glucose levels throughout pregnancy. As part of your routine care you are wearing a continuous glucose monitor. With this study we are investigating the impact of diet and lifestyle affects on glucose control throughout pregnancy as there is currently very little information on how diet and lifestyle affects glucose levels measures during pregnancy.

Therefore, as a first step, we want to monitor and study how 24-hour and mealtime glucose levels change in response to diet and across pregnancy in women with pre-existing type 1 or type 2 diabetes. Most information on glucose control and medical data will be requested via your medical records, if you give us permission for us to access your data. To decrease the burden of participation, we will use existing data as much as possible. However, to be able assess your diet and lifestyle during pregnancy, we will ask you to complete questionnaires via phone calls. These questionnaires are detailed below. None of the information obtained via the questionnaires will be shared with your clinical care team. This data on lifestyle will be anonymised and is solely for the purpose of this study.

For peer review only - http://bmjopen.bmj.com/site/about/guidelines.xhtml

UNIVERSITY OF LEEDS

The Leeds Teaching Hospitals NHS Trust

Why have I been invited?

We are approaching women with type 1 and type 2 diabetes, who are early in their pregnancy, to help us with this study. You do not have to take part – it is completely up to you — and does not affect the quality of care you receive from the NHS. Also, if you choose to take part and later decide to withdraw (for any or no reason), it will not affect your quality of care from the NHS.

What could my participation do?

By taking part in this study, you will help us to better understand how glucose levels change during pregnancy in women with type-1 and type-2 diabetes and the role of diet. By knowing this, we can design special diets and nutritional strategies to minimise the chances of babies being exposed to abnormal glucose levels and their risk of future health problems.

What would taking part involve?

Medical information. We ask you to give us consent to access selected parts of your medical record that reflect your general health and the health of your pregnancy (e.g., blood pressure, blood/urine test results, current medication, diabetes related pregnancy outcomes) and your diabetes health risks (e.g., age, body weight, ethnicity). Additionally, once you have given birth, your baby's birthweight, and any pregnancy complications will be copied from your medical records.

Urine samples. You will be providing urine samples regularly during pregnancy to your clinical team. Once they have been tested, rather than throwing them away we would like your consent to keep the remaining sample for future metabolic analysis.

Blood Samples. You will be having blood taken regularly during this pregnancy for your clinical care. On three of your routine visits to the Diabetes in Pregnancy Clinic we would like your consent to take an additional 10ml of blood for the study. We will store this to look at molecular and genetic markers that may be involved in metabolism and diabetes later. No infant blood samples are requested.

All samples will be stored at the University of Leeds in designated Human Tissue Act approved and compliant facilities.

Glucose Data. We ask for your consent for us to access your clinical glucose data throughout pregnancy. This will require no additional work on your part.

Lifestyle Questionnaires. On three occasions during your pregnancy, at ~10-12, ~18-20, and ~28-34, we will contact you at your convenience by phone or video call to complete some short questionnaires about your habitual physical activity, sleep quality / patterns and mealtimes. Also, following these three clinical visits, we will ask you to keep track of your diet for 3 days (2 weekdays and 1 weekend day) using an online dietary tracker called MyFood24. During the phone call, we will explain you how to use this dietary tracker. The phone calls will last not more than 30 minutes. The dietary tracker will take approximately 10 minutes per day to complete. This data will be anonymised and none of this data will be shared with your clinical care team.

Breakfast replacements. To gain more insight into mealtime glucose responses, we would like you to consider taking part in additional part of the study, where we will provide you with two different breakfast shakes to drink instead of your usual breakfast for 4 days on two separate occasions (one during your 2nd and one during your 3rd trimester). The two different breakfast shakes (e.g. Shake 1 and Shake 2) have the same amount of carbohydrate as that recommended during pregnancy, but one is designed to be absorbed slower, and the other faster so we can see how this affects your glucose measures on the continuous glucose monitor. Dependent on your randomization you will consume Shake 1 for two days followed by Shake 2 or vice versa. They are vegan friendly. The shakes will be delivered to your home with instructions for you to prepare.

For peer review only - http://bmjopen.bmj.com/site/about/guidelines.xhtml

You can of course still participate in the main study without having to take the breakfast shakes if you prefer.

This study will tell us, in detail previously unseen, how (mealtime) glucose changes across pregnancy and how diet can best be used to manage glucose levels and minimise maternal and infant health risks.

What are the possible risks of taking part?
Although we have designed the meals to not contain allergens and to release the same amount of glucose as you would usually eat for breakfast there is a possibility that you may experience an allergic reaction or higher glucose levels than normal after the standardised meal consumption. We will check that you have no allergies before taking part and ask you to contact the research team if you have these reactions to the meal. You will be advised to monitor and manage your blood glucose levels like you normally would and feel most comfortable with. However, if blood glucose levels surpass >18mmol/L for more than 90-minutes you are advised to administer a corrective dose of insulin or contact your GP/clinical care team. The meals are designed to minimize risk of hyperglycaemia. Blood samples are part of your routine clinical care and will be performed by qualified clinical staff, so any discomfort should be minimal.

What are the possible benefits of taking part?
There are no specific benefits to you of taking part, but participating in this study will give us important information about how to assess glucose in relation to personal characteristics, pregnancy outcomes, and newborn health. We anticipate that this will then help us to identify and develop new diet strategies to help women reduce their risk of small or large babies, stillbirths, pregnancy complications, and improve the long-term health of their children.

Further Information

What will happen if I don't want to carry on with the study?
You are free to withdraw at any time without explanation. If you decide not to carry on, it will not affect your care in anyway.

What if something goes wrong?
During the study, you will be covered by the Sponsor's Insurance, the University of Leeds is acting as Sponsor for this study. The University of Leeds has insurance cover in force, which meets claims against it and where those claims arise from the Universities own negligence in its role and activities relating to the study (and which is subject to the terms, conditions and exceptions of the relevant policy). Clinical negligence indemnification will rest with the participating NHS Trust under standard NHS arrangements.

If you are unhappy about any part of the study, you are encouraged to discuss this with the research team or with the Patient Assistance and Liaison Services (PALS) at your hospital. Normal legal processes are also open to you. We foresee minimal risks as most data will be collected from your routine clinical records and specific study risks are limited to questionnaires and meal replacements.

What will happen to my additional blood samples and urine sample?
Your blood and urine samples will be labelled with your unique study number and stored in freezers at the University of Leeds for longer term storage. Analysis of the samples will be undertaken for molecular and genetic factors that may contribute to a mother's metabolism, glucose control and babies growth. Only researchers directly involved in the study will have access to the samples. Results of samples analyses will only be used for the purpose of the research study.

How will we use information about you?
We will need to use information from you and from your medical records (including the continuous glucose monitoring data) for this research project. This information will include your initials/NHS number/date of birth/name/contact details. A member of your clinical care team will give you a unique random study

For peer review only - http://bmjopen.bmj.com/site/about/guidelines.xhtml

number, so that personal information cannot be traced back by members of the research team. Your consented medical data will be routinely stored on an electronic patient platform using your study number. Only authorised members of the research team will be able to access this platform and copy the data to secure university computers, this data will be stored for a maximum of 15 years, for analysis and writing up the results and also for authorised people to check your records to make sure that the research is being done properly. These people who do not need to know who you are will not be able to see your name or contact details. Your data will have a unique study number that will make you anonymous to the study team. We will keep all information about you safe and secure. Once we have finished the study, we will keep some of the data so we can check the results. All analyses and reports will be written in a way that no-one can work out that you took part in the study.

How will my information be kept confidential?
All information which is collected about you will be held securely and treated in accordance with the Regulation (EU) 2016/679 (General Data Protection Regulation) and the Data Protection Act 2018.

We will be using information collected by your local hospital from you and your medical records in order to undertake this study. No personal identifiable data will leave the NHS hospital without your consent; Data leaving the hospital will be labelled with your unique study number and will not have your name or any other identifying details on it. We refer to this as linked anonymised data as it is linked to you by a code. The code will only be known by key research team members. It will be kept securely.

Data which leaves the NHS Trust where you are being treated will be held securely in a database, operated by the data analysis team at the University of Leeds. This includes only linked anonymised study data and will not have your name or any other identifying details on it.

If you join the study, the data collected for the study, together with any relevant medical records, may be looked at by authorised persons from University of Leeds, the Research and Development Department of your local hospital and the Regulatory authorities to check the study is being carried out correctly. They all have a duty of confidentiality to you as a research participant.

Other third party researchers (e.g. universities, NHS organisations or companies involved in health and care research) may wish to access anonymised data (including samples) from this study in the future (anonymised data do not include names, addresses, or dates of birth, and it is not possible to identify individual participants from anonymised data). If this is the case, the Chief Investigator will ensure that the other researchers comply with legal, data protection and ethical guidelines. This may include research outside of the UK and EU and/or research that is commercial in nature. Your data will be stored securely for a period of 15 years after the end of the trial before being destroyed.

What are your choices about how your information is used?
If you withdraw consent during the study, no further data will be collected on you. However, any data (including samples) already collected by the research team may be retained and subsequently analysed for the purposes of the study. Your right to access, change or move your information are limited, as we need to manage your information in specific ways in order for the research to be reliable and accurate. To safeguard your rights we will use the minimum personally-identifiable information possible.

The University of Leeds as the Sponsor, is the data controller for this study. This means that we are responsible for looking after your information and using it properly.

The University of Leeds is the data processors for this study. The lawful basis for processing personal data collected in this study is that it is a task in the public interest. You can find out more about how we use your information at https://dataprotection.leeds.ac.uk/wp-content/uploads/sites/48/2019/02/Research-Privacy-Notice.pdf; and https://dataprotection.leeds.ac.uk/wp-content/uploads/sites/48/2019/09/HRA-

For peer review only - http://bmjopen.bmj.com/site/about/guidelines.xhtml

UNIVERSITY OF LEEDS

The Leeds Teaching Hospitals NHS Trust

transparency-wording.pdf by contacting University of Leeds Data Protection Officer's (e-mail: dpo@leeds.ac.uk).

What will happen to the study results?

The study is part of a PhD project and the result will be used for writing the doctorate thesis. The study results may be presented at meetings or published in scientific journals but individuals will not be identifiable. After the study has ended we will send a newsletter with the study results to your research team, which they will be able to share with you.

Who is organising and funding the research?

The research study has been primarily funded and sponsored by the University of Leeds with additional support from the Wellcome Trust (MZ). The Chief Investigators (Dr Michael Zulyniak and Professor Eleanor Scott) are University of Leeds researchers. Professor Scott is also one of the senior NHS consultants providing clinical care in the Diabetes Pregnancy Clinic.

Who has reviewed the study?

Before any research goes ahead it has to be checked by an Ethics Committee. This study has been reviewed by the Leeds East Research Ethics Committee.

What happens now if I agree to do the study?

The study procedures will be explained to you in more detail by the research team. You will be able to ask questions and voice any queries. If you agree to take part we will ask you to sign a consent form and complete screening questionnaires online to confirm eligibility, this will take approximately 10 minutes. If you are not eligible to participate, information provided prior to participation will be destroyed. The research team will then co-ordinate with you the dates for completing the lifestyle questionnaires, food dairy, and consuming the breakfast replacements

Please, contact the research team for more information:

Co-Investigator

Name: Cassy Dingena
Address: EC Stoner Building, University of Leeds, Woodhouse Ln, LS2 9JT
Email: fscd@leeds.ac.uk
Telephone: +316 27072821

Chief Investigators

Name: Dr Michael Zulyniak
Address: EC Stoner Building, University of Leeds, Woodhouse Ln, LS2 9JT
Email: m.a.zulyniak@leeds.ac.uk
Telephone: +44 (0)113 343 0685

Name: Professor Eleanor Scott
Address: Manny Cussins Diabetes Centre, St James's University Hospital, Leeds, LS9 7TF
Email: eleanor.scott9@nhs.net
Telephone 0113 2065014

For peer review only

For peer review only - http://bmjopen.bmj.com/site/about/guidelines.xhtml

UNIVERSITY OF LEEDS

NHS
The Leeds
Teaching Hospitals
NHS Trust

Supplementary section C: Consent form

INFORMED

Individualised patieNt care and treatment FOR MatErnal Diabetes

Understanding the glycaemic profile of maternal diabetes using continuous glucose monitoring: intensive glucose profiling to inform patient care and treatment

IRAS Project ID: 297276

Participant ID for this study:

Name of Researcher:

CONSENT FORM

Please **initial** all boxes that apply:

1. I confirm that I have read and understand the Participant Information Sheet dated ………………………. (Version……….) for the above study. I have had the opportunity to consider the information, ask questions and have had these answered satisfactorily. ☐

2. I understand that my participation is voluntary and that I am free to withdraw at any time without giving any reason, without my medical care or legal rights being affected in any way. ☐

3. I understand that relevant sections of my medical records of me and my baby, after their birth until either of us is discharged from hospital and data collected during this study may be looked at by members of the research team, from regulatory authorities or from the NHS Trust / sponsor, in this case the University of Leeds, where it is relevant to taking part in this research. I give permission for these individuals to have access to my records. ☐

4. I understand that my name will not be linked with the research materials, and I will not be identified or identifiable in the report or reports that result from the research. ☐

5. I agree for any unused urine (up to 2.5 mL) collected as routine care during this study to be stored securely long-term at University of Leeds for later analysis for research purposes only. These are considered a 'gift' from me, and may be used in relevant future research (in an anonymised form). I understand that this may involve co-operation with researchers outside of the UK) (*optional*). ☐

For peer review only - http://bmjopen.bmj.com/site/about/guidelines.xhtml

UNIVERSITY OF LEEDS

The Leeds Teaching Hospitals NHS Trust

6. I agree for any additional blood samples collected during this study to be stored securely long-term at University of Leeds for later analysis for research purposes only. These are considered a 'gift' from me, and may be used in relevant future research (in an anonymised form). I understand that this may involve co-operation with researchers outside of the UK) (*optional*).

    a. I give my consent for the taking of an additional blood sample for molecular analysis (*optional*).

    b. I give my consent for the taking of an additional blood sample for genetic/DNA analysis (*optional*).

7. I understand that the information collected about me and my baby may be used to support other ethically approved research in the future, and may be shared anonymously with other researchers. This may include research outside of the UK and EU and/or research that is commercial in nature.

8. I agree for my GP to be informed of my participation in this study.

9. I give consent to the research team to keep my contact details for them to contact me during and after the study (*optional*).

10. I am happy to be contacted about longer term follow up after ending of the study of myself or my baby (*optional*).

11. I agree to take part in the standardised meals study (*optional*).

12. I agree to take part in the study to the sections I have consented to.

Name of participant	Date	Signature
Name of person taking consent	Date*	Signature*

*To be signed and dated in the presence of the participant.

For peer review only - http://bmjopen.bmj.com/site/about/guidelines.xhtml

UNIVERSITY OF LEEDS

The Leeds Teaching Hospitals NHS Trust

Suplementary section D: Screening Questionnaire

INFORMED

Individualised patieNt care and treatment FOR MatErnal Diabetes

Understanding the glycaemic profile of maternal diabetes using continuous glucose monitoring: intensive glucose profiling to inform patient care and treatment

Screening questionnaire

Thank you for considering your participation in our study called PERFORMD. We would like to fill out this questionnaire, so we can assess your eligibility.

First name/s: ……………………………………………………………………………………………………

Last name: …………………………………………………………………………………………………

Address: …………………………………………………………………………………………………

Postcode:………………………………………………………………………………………………

Phone number:………………………………………………………………………………………

E-mail address:……………………………………………………………………………………

Date of birth: ……………………………………………………………………………………

General Practitioner (GP)/Family doctor

Name:………………………………………………………………………………………

Address:……………………………………………………………………………………

Postcode:…………………………………………………………………………………

Phone number:……………………………………………………………………………

For peer review only - http://bmjopen.bmj.com/site/about/guidelines.xhtml

UNIVERSITY OF LEEDS

The Leeds Teaching Hospitals NHS Trust

Medical specialist (if applicable)

Name: ………

Address:……

Postcode: ………………………………………………………………………………………………

Phone number: ………………………………………………………………………………………………

1. What is your ethnic origin?
   ☐ White (Caucasian)
   ☐ Black (African-American)
   ☐ Asian
   ☐ Mixed

2. What is the highest educational qualification that you have?
   ☐ No qualifications
   ☐ Achieved GCSE grades D-G, NVQ Level 1, Skills For Life level 1, BTEC-award Certificate or diploma level 1, OCR National
   ☐ GCSE grades A*-C, NVQ Level 2, BTEC Award Certificate OR diploma level 2
   ☐ AS & A level, NVQ Level 3, Advanced Extension award, International Baccalaureate, OCR National
   ☐ NVQ Level 4, BTEC Professional award, Certificate of Higher Education
   ☐ BTEC Award Advanced professional / Bachelors Degree / Graduate Diploma
   ☐ University Masters Degree / Postgraduate diploma / NVQ Level 5 / BTEC Advanced Professional Award Certificate and Diploma level 7
   ☐ Doctorate (e.g. PhD, DClin.)

For peer review only - http://bmjopen.bmj.com/site/about/guidelines.xhtml

UNIVERSITY OF LEEDS

The Leeds Teaching Hospitals NHS Trust

3. What do you do?
   ☐ I am a student
   ☐ I am employed
   ☐ I am self employed
   ☐ I am a housewife, househusband
   ☐ I am unemployed

   ☐ I am unable to work (e.g. due to a disability)

   ☐ I am retired
   ☐ I do something else (e.g. volunteering), namely...

4. When is your expected due date?
   If you do not know exactly, try to estimate it as well as possible.

   |__|__| |__|__| |__|__|__|__|
   Day Month Year

5. How long have you been pregnant? (weeks)
   If you do not know exactly, try to estimate it as well as possible.

   ....

6. Do you have children?
   ☐ Yes, I have …. child(ren).
   ☐ No

7. Do you have a singleton pregnancy?
   ☐ Yes, I am expecting an single child
   ☐ No, I am expecting twins, triplets etc.

8. Would you say your general health is…..?
   ☐ Excellent
   ☐ Very Good
   ☐ Good
   ☐ Fair
   ☐ Poor
   ☐ Don't know/Not sure
   ☐ I rather not say

9. Are you diagnosed with diabetes (Type 1, Type 2, Gestational diabetes)?
   ☐ Yes, Please specify type of diabetes ....
   ☐ No

10. For how long have you been diagnosed with diabetes?
    If you do not know exactly, try to estimate it as well as possible.

For peer review only - http://bmjopen.bmj.com/site/about/guidelines.xhtml
For peer review only

UNIVERSITY OF LEEDS

The Leeds Teaching Hospitals NHS Trust

Please, specify in years ....

11. Did you take any medication (including diabetes medication) in the last month?
☐ No
☐ Yes

If yes, please specify which medication, the dose and how many times a day.

For example: Routine: Omeprazole 40mg once a day for 7 days or one-time treatment: amoxicillin 500mg once a for 7 days.

....

....

....

....

....

12. Do you currently smoke or use e-cigarettes?
☐ Yes - smoke cigarettes
☐ Yes - smoke cigars
☐ Yes - use e- cigarettes
☐ No, I quit smoking → go on to question 22
☐ No, I never smoked → go on to question 23

13. How many cigarettes/cigars do you normally smoke?
☐ 1-5 each day

☐ 6-10 each day
☐ More than 10 each day
☐ None, I smoke pipe or vape

14. What year did you start and quit smoking?
Started: (yyyy) |__|__|__|__|
Quit: (yyyy) |__|__|__|__|

For peer review only - http://bmjopen.bmj.com/site/about/guidelines.xhtml
For peer review only

UNIVERSITY OF LEEDS

NHS
The Leeds Teaching Hospitals
NHS Trust

15. How many units of alcohol did you normally (before pregnancy) consume during the week? (A Guide to the number of units of alcohol in some typical alcoholic drinks is provided). https://www.nhs.uk/Livewell/alcohol/Pages/alcohol-units.aspx

☐ I do not use any alcohol
☐ Less than 1 unit a week
☐ 1 - 5 units a week
☐ 6 - 7 units a week
☐ 8 - 15 units a week
☐ 16 - 30 units a week
☐ More than 30 units a week

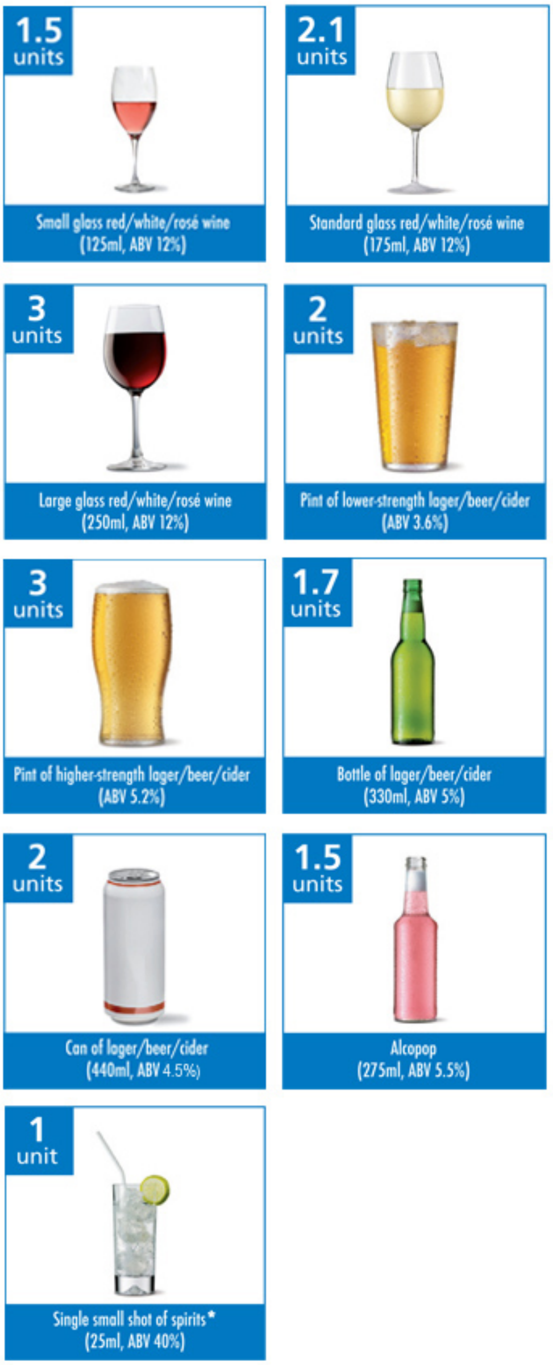

For peer review

For peer review only - http://bmjopen.bmj.com/site/about/guidelines.xhtml

16. Do you have any food allergies?
☐ No
☐ Yes

If yes, please tick any allergies that apply below:

☐ Tree Nuts (e.g. walnuts, almonds, pine nuts, brazil nuts, and pecans)
☐ Peanuts
☐ Cow's milk
☐ Other milk
☐ Eggs
☐ Wheat
☐ Barley
☐ Oats
☐ Molluscs
☐ Lupin
☐ Sesame
☐ Sulphites
☐ Soy
☐ Mustard
☐ Celery
☐ Fish, shellfish and crustaceans
☐ Other, namely...

17. Do you use any dietary supplements? (i.e. vitamin supplements, minerals, fibres or probiotics). Examples of commonly used probiotics include:
- Actimel drink
- Activia yogurt
- Benecol yogurt drink
- Yakult drink
- Arla Skyr yogurt drink
- Creamier bio-live Irish yogurts
- Probiotics from a local chemist or herbalist (e.g. Acidophilus Capsules)

☐ No
☐ Yes

If yes, what products have you used: How often do you use them (e.g. once per day):

....

....

....

For peer review only - http://bmjopen.bmj.com/site/about/guidelines.xhtml

18. Do you have internet access on a computer, tablet or smartphone at home?
   ☐ Yes
   ☐ No

19. Are you currently participating in any other research studies?
   ☐ Yes
   ☐ No

   If yes, please specify which study and give brief explanation.

   ....

   ....

   ....

   ....

   ....

Can we keep this information on file and contact you about future studies? Yes / No

For peer review only

For peer review only - http://bmjopen.bmj.com/site/about/guidelines.xhtml

Suplementary section E: Medical history questionnaire

INFORMED

Individualised patieNt care and treatment FOR MatErnal Diabetes

Understanding the glycaemic profile of maternal diabetes using continuous glucose monitoring: intensive glucose profiling to inform patient care and treatment

For peer review only

Medical History Questionnaire

1. Do you currently have or have you had history of any of the following diseases?

If you have selected yes for any of the following conditions, please indicate which (if any) you are currently receiving treatment for? Please also state whether the condition limits you in your daily activities.

	Have you/ are you currently suffering from any of the following?		Are you receiving treatment for this disease?		Does this disease limit you in your daily activities?	
**Diseases**	*No*	*Yes*	*No*	*Yes*	*No*	*Yes*
Disease of heart/coronary arteries (angina pectoris, heart attack)						
Hypertension						
Lung disease						
Asthma						
Diabetes						
Stomach ulcers or other stomach disorders						
Kidney disease						
Liver disease						
Anaemia or any other blood disease						
Cancer						
Depression						
Eating disorder (e.g. anorexia/bulimia)						
Chronic fatigue syndrome (CFS)						

For peer review only - http://bmjopen.bmj.com/site/about/guidelines.xhtml

Stroke						
Gastrointestinal disease						
Oesophageal disease						
Thyroid disease						
High cholesterol						

If you are suffering from any other medical problems, please specify below:	*No*	*Yes*	*No*	*Yes*
……………………………………………………………………………………… ………………………………				
……………………………………………………………………………………… ………………………………				
……………………………………………………………………………………… ………………………………				

2. In the past, have you had any major abdominal surgery (please provide dates)?

Not had any major abdominal surgery in the past

Yes, had major abdominal surgery in the past, namely:

Laparoscopic (or key-hole): appendectomy Cholecystectomy

Open surgery: appendectomy Cholecystectomy

Other abdominal surgery, namely: ……………………………………

Date/s of any surgery:……………………………………………………………………………

Appendectomy: removal of the appendix

Cholecystectomy: removal of the gall bladder

For peer review only - http://bmjopen.bmj.com/site/about/guidelines.xhtml

Suplementary section F: Modified Pregnancy Physical Activity Questionnaire (PPAQ)

INFORMED

Individualised patieNt care and treatment FOR MatErnal Diabetes

Understanding the glycaemic profile of maternal diabetes using continuous glucose monitoring: intensive glucose profiling to inform patient care and treatment

---

Physical activity questionnaire

During this trimester, how much time do you usually spend on:

1. Preparing meals (cook, set table, wash dishes)

None

Less than ½ hour per day

½ to almost 1 hour per day

1 to almost 2 hour per day

2 to almost 3 hour per day

3 or more hours per day

2. Taking care of an older adult

None

Less than ½ hour per day

½ to almost 1 hour per day

1 to almost 2 hour per day

2 to almost 3 hour per day

3 or more hours per day

3. Sitting and using a computer or writing, <u>not for work</u>

None

Less than ½ hour per day

For peer review only - http://bmjopen.bmj.com/site/about/guidelines.xhtml

½ to almost 1 hour per day

1 to almost 2 hour per day

2 to almost 3 hour per day

3 or more hours per day

4. Sitting at work or in class → If the participant does not work or study, skip to question 7.

None

Less than ½ hour per day

½ to almost 1 hour per day

1 to almost 2 hour per day

2 to almost 3 hour per day

3 or more hours per day

5. Standing or walking at work while carrying things (heavier than a 1 gallon milk jug)

None

Less than ½ hour per day

½ to almost 1 hour per day

1 to almost 2 hour per day

2 to almost 3 hour per day

3 or more hours per day

6. Standing or walking at work while not carrying anything

None

Less than ½ hour per day

½ to almost 1 hour per day

1 to almost 2 hour per day

2 to almost 3 hour per day

3 or more hours per day

7. Sitting and reading, talking or on the phone, not for work

None

For peer review only - http://bmjopen.bmj.com/site/about/guidelines.xhtml

Less than ½ hour per day

½ to almost 1 hour per day

1 to almost 2 hour per day

2 to almost 3 hour per day

3 or more hours per day

8. Watching TV or video

None

Less than ½ hour per day

½ to almost 1 hour per day

1 to almost 2 hour per day

2 to almost 3 hour per day

3 or more hours per day

9. Light cleaning (make beds, laundry, ironing, putting things away)

None

Less than ½ hour per day

½ to almost 1 hour per day

1 to almost 2 hour per day

2 to almost 3 hour per day

3 or more hours per day

10. Heavier cleaning (vacuum, mop, sweep, wash windows)

None

Less than ½ hour per day

½ to almost 1 hour per day

1 to almost 2 hour per day

2 to almost 3 hour per day

3 or more hours per day

11. Shopping (for food, clothes, or other)

For peer review only

For peer review only - http://bmjopen.bmj.com/site/about/guidelines.xhtml

None

Less than ½ hour per day

½ to almost 1 hour per day

1 to almost 2 hour per day

2 to almost 3 hour per day

3 or more hours per day

12. Gardening

None

Less than ½ hour per day

½ to almost 1 hour per day

1 to almost 2 hour per day

2 to almost 3 hour per day

3 or more hours per day

Please, fill out the next section if you have children. If you do not take care of children, you do not need to complete the this section.

During this trimester, taking care of children, how much time do you usually spend on:

13. Dressing, bathing, feeding children while you are sitting

None

Less than ½ hour per day

½ to almost 1 hour per day

1 to almost 2 hour per day

2 to almost 3 hour per day

3 or more hours per day

14. Dressing, bathing, feeding children while you are standing

None

Less than ½ hour per day

½ to almost 1 hour per day

For peer review only - http://bmjopen.bmj.com/site/about/guidelines.xhtml

1 to almost 2 hour per day

2 to almost 3 hour per day

3 or more hours per day

15. Playing with children while you are standing or sitting

None

Less than ½ hour per day

½ to almost 1 hour per day

1 to almost 2 hour per day

2 to almost 3 hour per day

3 or more hours per day

16. Playing with children while you are walking or running

None

Less than ½ hour per day

½ to almost 1 hour per day

1 to almost 2 hour per day

2 to almost 3 hour per day

3 or more hours per day

17. Carrying children

None

Less than ½ hour per day

½ to almost 1 hour per day

1 to almost 2 hour per day

2 to almost 3 hour per day

3 or more hours per day

During this trimester, how much time do you usually spend on exercising:

18. Walking to go places (such as to the bus, work, visiting). Not for fun or exercise

None

For peer review only - http://bmjopen.bmj.com/site/about/guidelines.xhtml

Less than ½ hour per day

½ to almost 1 hour per day

1 to almost 2 hour per day

2 to almost 3 hour per day

3 or more hours per day

19. Walking for fun or exercise

None

Less than ½ hour per day

½ to almost 1 hour per day

1 to almost 2 hour per day

2 to almost 3 hour per day

3 or more hours per day

20. Jogging

None

Less than ½ hour per day

½ to almost 1 hour per day

1 to almost 2 hour per day

2 to almost 3 hour per day

3 or more hours per day

21. Prenatal exercise class

None

Less than ½ hour per day

½ to almost 1 hour per day

1 to almost 2 hour per day

2 to almost 3 hour per day

3 or more hours per day

22. Doing other things for fun or exercise (such as swimming or dancing)?

For peer review only - http://bmjopen.bmj.com/site/about/guidelines.xhtml
For peer review only

None

Less than ½ hour per day

½ to almost 1 hour per day

1 to almost 2 hour per day

2 to almost 3 hour per day

3 or more hours per day

For peer review only

For peer review only - http://bmjopen.bmj.com/site/about/guidelines.xhtml

Suplementary section G: Modified Leeds Sleep Evaluation Questionnaire (LSEQ)

INFORMED

Individualised patieNt care and treatment FOR MatErnal Diabetes

Understanding the glycaemic profile of maternal diabetes using continuous glucose monitoring: intensive glucose profiling to inform patient care and treatment

Sleep quality questionnaire

How would you describe the way you currently fall asleep in comparison to usual?

1. More difficult than usual -------------- Easier than usual

2. Slower than usual -------------- More quickly than usual

3. I feel less sleepy than usual -------------- More sleepy than usual

How would you describe the quality of your sleep compared to normal sleep?

4. More restless than usual -------------- Calmer than usual

5. With more wakeful periods than usual ------------- With less wakeful periods

How would you describe your awakening in comparison to usual?

6. More difficult than usual -------------- Easier than usual

7. Requires a period of time longer than usual -------------- Shorter than usual

How do you feel when you wake up?

8. Tired -------------- Alert

How do you feel now?

9. Tired -------------- Alert

How would you describe your balance and co-ordination upon awakening?

10. More disrupted than usual -------------- Less disrupted than usual]

For peer review only

For peer review only - http://bmjopen.bmj.com/site/about/guidelines.xhtml

STANDARD PROTOCOL ITEMS: RECOMMENDATIONS FOR INTERVENTIONAL TRIALS

SPIRIT 2013 Checklist: Recommended items to address in a clinical trial protocol and related documents*

Section/item	Item No	Description	Pg
**Administrative information**			
Title	1	Descriptive title identifying the study design, population, interventions, and, if applicable, trial acronym	X
Trial registration	2a	Trial identifier and registry name. If not yet registered, name of intended registry	X
	2b	All items from the World Health Organization Trial Registration Data Set	X
Protocol version	3	Date and version identifier	X
Funding	4	Sources and types of financial, material, and other support	X
Roles and responsibilities	5a	Names, affiliations, and roles of protocol contributors	X
	5b	Name and contact information for the trial sponsor	X
	5c	Role of study sponsor and funders, if any, in study design; collection, management, analysis, and interpretation of data; writing of the report; and the decision to submit the report for publication, including whether they will have ultimate authority over any of these activities	X
	5d	Composition, roles, and responsibilities of the coordinating centre, steering committee, endpoint adjudication committee, data management team, and other individuals or groups overseeing the trial, if applicable (see Item 21a for data monitoring committee)	X
**Introduction**			
Background and rationale	6a	Description of research question and justification for undertaking the trial, including summary of relevant studies (published and unpublished) examining benefits and harms for each intervention	X
	6b	Explanation for choice of comparators	X
Objectives	7	Specific objectives or hypotheses	X

For peer review only - http://bmjopen.bmj.com/site/about/guidelines.xhtml

Trial design	8	Description of trial design including type of trial (eg, parallel group, crossover, factorial, single group), allocation ratio, and framework (eg, superiority, equivalence, noninferiority, exploratory)	X
**Methods: Participants, interventions, and outcomes**			
Study setting	9	Description of study settings (eg, community clinic, academic hospital) and list of countries where data will be collected. Reference to where list of study sites can be obtained	X
Eligibility criteria	10	Inclusion and exclusion criteria for participants. If applicable, eligibility criteria for study centres and individuals who will perform the interventions (eg, surgeons, psychotherapists)	X
Interventions	11a	Interventions for each group with sufficient detail to allow replication, including how and when they will be administered	X
	11b	Criteria for discontinuing or modifying allocated interventions for a given trial participant (eg, drug dose change in response to harms, participant request, or improving/worsening disease)	X
	11c	Strategies to improve adherence to intervention protocols, and any procedures for monitoring adherence (eg, drug tablet return, laboratory tests)	X
	11d	Relevant concomitant care and interventions that are permitted or prohibited during the trial	X
Outcomes	12	Primary, secondary, and other outcomes, including the specific measurement variable (eg, systolic blood pressure), analysis metric (eg, change from baseline, final value, time to event), method of aggregation (eg, median, proportion), and time point for each outcome. Explanation of the clinical relevance of chosen efficacy and harm outcomes is strongly recommended	X
Participant timeline	13	Time schedule of enrolment, interventions (including any run-ins and washouts), assessments, and visits for participants. A schematic diagram is highly recommended (see Figure)	X
Sample size	14	Estimated number of participants needed to achieve study objectives and how it was determined, including clinical and statistical assumptions supporting any sample size calculations	X

Recruitment	15	Strategies for achieving adequate participant enrolment to reach target sample size	X

Methods: Assignment of interventions (for controlled trials)

Allocation:

Sequence generation	16a	Method of generating the allocation sequence (eg, computer-generated random numbers), and list of any factors for stratification. To reduce predictability of a random sequence, details of any planned restriction (eg, blocking) should be provided in a separate document that is unavailable to those who enrol participants or assign interventions	X
Allocation concealment mechanism	16b	Mechanism of implementing the allocation sequence (eg, central telephone; sequentially numbered, opaque, sealed envelopes), describing any steps to conceal the sequence until interventions are assigned	X
Implementati on	16c	Who will generate the allocation sequence, who will enrol participants, and who will assign participants to interventions	X
Blinding (masking)	17a	Who will be blinded after assignment to interventions (eg, trial participants, care providers, outcome assessors, data analysts), and how	X
	17b	If blinded, circumstances under which unblinding is permissible, and procedure for revealing a participant's allocated intervention during the trial	X

Methods: Data collection, management, and analysis

Data collection methods	18a	Plans for assessment and collection of outcome, baseline, and other trial data, including any related processes to promote data quality (eg, duplicate measurements, training of assessors) and a description of study instruments (eg, questionnaires, laboratory tests) along with their reliability and validity, if known. Reference to where data collection forms can be found, if not in the protocol	X
	18b	Plans to promote participant retention and complete follow-up, including list of any outcome data to be collected for participants who discontinue or deviate from intervention protocols	X
Data management	19	Plans for data entry, coding, security, and storage, including any related processes to promote data quality (eg, double data entry; range checks for data values). Reference to where details of data management procedures can be found, if not in the protocol	X

For peer review only - http://bmjopen.bmj.com/site/about/guidelines.xhtml

Statistical methods	20a	Statistical methods for analysing primary and secondary outcomes. Reference to where other details of the statistical analysis plan can be found, if not in the protocol	X
	20b	Methods for any additional analyses (eg, subgroup and adjusted analyses)	X
	20c	Definition of analysis population relating to protocol non-adherence (eg, as randomised analysis), and any statistical methods to handle missing data (eg, multiple imputation)	X
**Methods: Monitoring**			
Data monitoring	21a	Composition of data monitoring committee (DMC); summary of its role and reporting structure; statement of whether it is independent from the sponsor and competing interests; and reference to where further details about its charter can be found, if not in the protocol. Alternatively, an explanation of why a DMC is not needed	X
	21b	Description of any interim analyses and stopping guidelines, including who will have access to these interim results and make the final decision to terminate the trial	X
Harms	22	Plans for collecting, assessing, reporting, and managing solicited and spontaneously reported adverse events and other unintended effects of trial interventions or trial conduct	X
Auditing	23	Frequency and procedures for auditing trial conduct, if any, and whether the process will be independent from investigators and the sponsor	X
**Ethics and dissemination**			
Research ethics approval	24	Plans for seeking research ethics committee/institutional review board (REC/IRB) approval	X
Protocol amendments	25	Plans for communicating important protocol modifications (eg, changes to eligibility criteria, outcomes, analyses) to relevant parties (eg, investigators, REC/IRBs, trial participants, trial registries, journals, regulators)	X
Consent or assent	26a	Who will obtain informed consent or assent from potential trial participants or authorised surrogates, and how (see Item 32)	X
	26b	Additional consent provisions for collection and use of participant data and biological specimens in ancillary studies, if applicable	X

For peer review only - http://bmjopen.bmj.com/site/about/guidelines.xhtml

Confidentiality	27	How personal information about potential and enrolled participants will be collected, shared, and maintained in order to protect confidentiality before, during, and after the trial	X
Declaration of interests	28	Financial and other competing interests for principal investigators for the overall trial and each study site	X
Access to data	29	Statement of who will have access to the final trial dataset, and disclosure of contractual agreements that limit such access for investigators	X
Ancillary and post-trial care	30	Provisions, if any, for ancillary and post-trial care, and for compensation to those who suffer harm from trial participation	X
Dissemination policy	31a	Plans for investigators and sponsor to communicate trial results to participants, healthcare professionals, the public, and other relevant groups (eg, via publication, reporting in results databases, or other data sharing arrangements), including any publication restrictions	X
	31b	Authorship eligibility guidelines and any intended use of professional writers	X
	31c	Plans, if any, for granting public access to the full protocol, participant-level dataset, and statistical code	X
**Appendices**			
Informed consent materials	32	Model consent form and other related documentation given to participants and authorised surrogates	X
Biological specimens	33	Plans for collection, laboratory evaluation, and storage of biological specimens for genetic or molecular analysis in the current trial and for future use in ancillary studies, if applicable	X

*It is strongly recommended that this checklist be read in conjunction with the SPIRIT 2013 Explanation & Elaboration for important clarification on the items. Amendments to the protocol should be tracked and dated. The SPIRIT checklist is copyrighted by the SPIRIT Group under the Creative Commons "Attribution-NonCommercial-NoDerivs 3.0 Unported" license.

For peer review only - http://bmjopen.bmj.com/site/about/guidelines.xhtml

BMJ Open

Protocol for the INFORMED (Individualised Patient Care and Treatment for Maternal Diabetes): A randomised controlled trial embedded within routine care

Journal:	*BMJ Open*
Manuscript ID	bmjopen-2022-065388.R1
Article Type:	Protocol
Date Submitted by the Author:	11-Jan-2023
Complete List of Authors:	Dingena, Cassy; University of Leeds, Food Science and Nutrition<br>Mahendra, Anvesha; University of Leeds, Food Science and Nutrition<br>Holmes, Melvin; University of Leeds, Food Science and Nutrition<br>Clement, Naomi S; University of Leeds, Medicine<br>Scott, Eleanor; University of Leeds, Division of Clinical and Population Sciences, Leeds Institute of Cardiovascular and Metabolic Medicine<br>Zulyniak, Michael; University of Leeds
**Primary Subject Heading**:	Diabetes and endocrinology
Secondary Subject Heading:	Diabetes and endocrinology, Epidemiology, Nutrition and metabolism, Obstetrics and gynaecology
Keywords:	NUTRITION & DIETETICS, Diabetes in pregnancy < DIABETES & ENDOCRINOLOGY, General diabetes < DIABETES & ENDOCRINOLOGY

SCHOLARONE™
Manuscripts

1

TITLE: Protocol for the INFORMED (Individualised Patient Care and Treatment for Maternal Diabetes): A randomised controlled trial embedded within routine care

AUTHORS: Cassy Dingena[1], Anvesha Mahendra[1], Melvin Holmes[1], Naomi S Clement[2], Eleanor Scott [3] and Michael A. Zulyniak[1*]

AUTHOR AFFILIATIONS:

[1] Nutritional Epidemiology Group, School of Food Science and Nutrition, University of Leeds, Leeds, UK.

[2] School of Medicine, University of Leeds, UK.

[3] Division of Clinical and Population Sciences, Leeds Institute of Cardiovascular and Metabolic Medicine, University of Leeds, Leeds, UK.

* CORRESPONDING AUTHOR: Michael A. Zulyniak, Nutritional Epidemiology Group, School of Food Science and Nutrition, University of Leeds, Leeds, LS2 9JT. Email: m.a.zulyniak@leeds.ac.uk. Ph. +44(0)1133430685

WORD COUNT: 3650

KEYWORDS: pregnancy, CGM, glucose, dysglycemia, RCT, nutrition

For peer review only

2

ABSTRACT

Introduction: Diabetes in pregnancy presents a unique physiological challenge to manage glycaemia, while maintaining adequate nourishment for the growing foetus. Women with diabetes who become pregnant are at greater risk of adverse maternal and newborn outcomes, compared to non-diabetic women. Evidence suggests that control of (post-prandial) glycaemia is key to manage maternal and offspring health but it is not yet clear (i) how diet and lifestyle moderate these shifts across the full duration of pregnancy or (ii) what aspects of maternal and offspring health are associated with dysglycemia.

Methods and Analysis: To investigate these gaps, a cross-over randomised clinical trial has been embedded within routine clinical care. Seventy-six pregnant women in their 1st trimester with T1D/T2D (with or without medication) attending their routine antenatal appointments at NHS Leeds Teaching Hospitals will be recruited. Following informed consent, data on women's health, glycaemia, pregnancy, and delivery will be shared by the NHS with researchers. At each visit in the 1st (10-12 weeks), 2nd (18-20 weeks), and 3rd (28-34 weeks) trimester participants will be asked for consent to: (i) lifestyle and diet questionnaires, (ii) blood for research purposes, and (iii) analysis of urine collected at clinical visits. Additionally, participants will be asked to consume two blinded meals in duplicate in 2nd and 3rd trimester. Glycemia will be assessed by continuous glucose monitoring as part of routine care. The primary outcome is the effect of experimental meals (high versus low protein) on post-prandial glycaemia. Secondary outcomes include (i) the association between dysglycemia and maternal and newborn health, and (ii) the association between maternal metabolic profiles in early pregnancy with dysglycemia in later pregnancy.

Ethics And Dissemination: The Leeds East Research Ethics Committee and NHS approved the study. Results will be published in peer-reviewed journals and disseminated to participants and the wider public.

ISRCTN: 57579163 (Protocol CPMS 50813)

REC: 21/NE/0196.

For peer review only

3

STRENGTHS AND LIMITATIONS OF THIS STUDY

- The analyses of response to meals alongside repeated measures of blood and urine metabolite profiles, will offer insights into distinct shifts in metabolism during pregnancy in women with type-1 and type-2 diabetes, and their association with maternal and newborn health at delivery.
- The trial is embedded within standard clinical care and uses routine data and biological samples collected by health services to minimise (i) participant burden, and (ii) non-essential participant contact, which reduces risk of bias and will permit the study to continue even with the re-introduction of public restrictions.
- As with all observational data, participant recall data (sleep, physical activity, and diet) are subject to social desirability bias but the inclusion of repeated and complementary measures (i.e., metabolite) will allow this to be evaluated in future analysis.
- INFORMED is being conducted with the National Health Service (NHS) of the UK; therefore, its results may not be directly generalizable to other nations or government health services.

INTRODUCTION

Background & Scope

Pregnancy naturally induces a state of mild insulin resistance (IR) to shuttle more nutrients to the growing baby; however, in women with diabetes in pregnancy (DIP), excessive insulin resistance and persistent hyperglycaemia increases the risk of adverse pregnancy outcomes[1-4]. Globally, the prevalence of DIP is on the rise, affecting ~17% of all pregnancies[4 5]. Compared to women without diabetes, women with DIP are at elevated risk of pre-eclampsia, preterm delivery, and mortality, while their offspring are at increased risk of unhealthy weight (<2.5 kgs or >4.5 kgs.), dysglycemia, injuries at birth, and higher risk of type 2 diabetes mellitus (T2DM) and cardiovascular disease (CVD) in later life[1 5 6].

Post-prandial glycaemic control is important for healthy pregnancy outcomes[1 6]. Evidence supports a healthy diet and lifestyle – that includes wholegrains, fruits and vegetables, and regular physical activity – as the cornerstone for managing DIP, which is effective in 70-85% of women with DIP[7-9]. NICE UK guidelines primarily focus on improving carbohydrate quality by including more low glycaemic index (GI) foods as part of a balanced diet including wholegrains, fruits and vegetables to manage glycaemia during pregnancy [7]. Although low GI diets do support the management of mean glucose levels, their effect on reducing episodes of

For peer review only

hypo- and hyperglycaemia and ability to reduce maternal and offspring risk of complications is not clearly established[10]. Alternatively, emerging evidence in preclinical and human studies, suggest that the amount of maternal protein intake can improve management of dysglycemia in DIP [(11)] but it's effect on metabolism and 24-hr dysglycemia in pregnancy is unknown. Finally, some women find it challenging to consistently follow a balanced diet, due to barriers such as availability, accessibility and affordability of healthy foods, lack of time and cooking skills [7 & 9]; therefore, a cost-effective nutritious meal replacement may be useful for supporting healthy eating habits. Most recently, we noted that 'morning' is when pregnant women with diabetes struggle most to manage glucose levels within a healthy range,[10] suggesting that breakfast may be a particularly important point in the day to offer support for managing dysglycemia.

Continuous glucose monitors (CGMs) are becoming routinely used in the UK National Health Service in perinatal clinical settings for women with DIP[1]. The unobtrusive patches record an individual's glucose every 5 mins for up to 14 days and offer quantitative information to identify interstitial glucose deviations over a 24-hr period. By measuring glucose continuously over hours and days, a more complete representation of dysglycemia can be modelled and offer novel insight regarding the parameters that drive and associate with dysglycaemia, and their impact on maternal and offspring health[11].

Previous studies[12 13] have uncovered new associations and identified novel points of interest for managing dysglycemia during pregnancy and mediating health risks. However, our ability to inform new strategies to manage these new areas of concern are limited by our understanding of the contribution of biological, lifestyle, and environment exposures on dysglycemia in early, mid, and late pregnancy and their moderating effect on maternal and offspring health. To address this gap in current understanding, this study aims to investigate the effect of breakfast meal replacements and dietary protein on glucose variability in pregnancy in women with pre-existing Type 1 or Type 2 Diabetes.

Aim And Objectives

Our overall aim is to investigate post-prandial CGM glucose profiles throughout the course of the pregnancy and how they are associated with personal (lifestyle) characteristics and physiological parameters. Our primary research objective is to assess the effect of easy-to-prepare meals and dietary protein on dysglycemia over the course of pregnancy. Secondary research objectives include (i) the association between dysglycemia and maternal and newborn

For peer review only

health, and (ii) the association between maternal metabolic profiles in early pregnancy with dysglycemia in later pregnancy.

METHODOLOGY AND ANALYSIS

Participants

Women with type 1 and 2 diabetes mellitus (T1DM and T2DM) during pregnancy in their 1st trimester will be recruited from the DIP antenatal clinics at Leeds Teaching Hospitals NHS Trust (LTHT). Women will be approached by their direct clinical care team and given a study information flyer and invited to contact the research team (phone or email) if they are interested to participate or if they wish to discuss the study in more detail. Women expressing interest at the end of the initial meeting will be emailed a participant information sheet and a web link to a secure electronic informed consent. Once a secure electronic signature is provided, the participant's eligibility will be assessed according to study inclusion and exclusion criteria.

Sample size using power calculation

CGM data provides numerous metrics to offer unique insight into variations and deviations of glucose levels over time – area under the curve (AUC), mean glucose, coefficient of variation (CV) of glucose, mean amplitude of glucose excursions (MAGE), and time in range (TIR)[14]. Additionally, there is no current evidence regarding the effect of diet composition and glycaemic load on metrics of continuous glucose monitoring (CGM) in pregnant women with diabetes throughout the duration of pregnancy. Therefore, we have elected to focus on AUC as the primary metric because it is easily-interpretable and commonly used to quantify post-prandial glycemia. Evidence from Fabricatore et al (2011)[15] demonstrated a significant association ($p<0.05$) between self-reported glycemic index (GI) and measures of CGM (including AUC, mean glucose, and % time hyperglycemic) in a clinical trial of 21 women and 5 men with type-2 diabetes. Assuming similar effect sizes between GI (per unit) and AUC glucose (β=0.36 mg/dl/min; R^2=0.38), mean glucose (β=0.02 mol/L; R^2=0.38), and time spent > 10 mol /L blood glucose (β=0.41%; R^2=0.36), we will have sufficient power (power = 0.90) to detect a significant pairwise effect of GI on these parameters with 63 participants. Another study by Law et al (2019) suggested that this will provide sufficient power (power = 0.90) to compare subgroups of participants (stratified by BMI, age, type of diabetes) and test for significant differences (of a minimum effect size) in AUC glucose (± 61 mmol/L/min), mean glucose (± 0.5 mmol/L), and % time hyperglycaemic (± 3.7 %)[16]. Finally, given the comparable

For peer review only

proportions of women reported to be of white European vs non-white European descent (57% vs 43%) or diet vs diet+ medication (46% vs 54%) we also anticipate having adequate power to compare these confounders of glycaemic response. To account for attrition, we will increase our recruitment target by 20% above our calculated, suggesting a target sample size of 76 recruited women. We have allocated ~6 months to recruit 76 women (at 10-12 weeks gestation) and ~15 months for study completion (i.e., final delivery). All power analysis were performed using G*Power (v3.1)[17].

Study participation inclusion and exclusion criteria

All pregnant women over the age of 18, with pre-existing T1DM or T2DM, in their 1st trimester and a singleton pregnancy, will be considered for the study. Women without T1DM or T2DM that develop gestational diabetes will not be eligible for the study because they would not be diagnosed until 26-28 weeks gestation are not currently offered CGM. Exclusion criteria include: (i) inability to understand English sufficiently to read the participant information sheet (PIS) and provide consent (**Supplementary Material, A-C)**; (ii) mental and/or psychological disorders that undermine informed consent; (iii) cancer, digestive tract disorders; (iv) lack of internet access on a computer or tablet at home. Detailed exclusion criteria in **Table 1** (Study screening questionnaires in **Supplementary Material, D and E**).

Table 1: Study participant inclusion and exclusion criteria

INCLUSION CRITERIA	EXCLUSION CRITERIA
Women aged 18-45 years	Women under 18 or above 45 years of age
Singleton pregnancy	Multiple pregnancy
Women in the first trimester of pregnancy	Foetal congenital abnormality
Previously diagnosed with type 1 or type 2 diabetes mellitus.	No diagnosis of diabetes
	Diagnosis of gestational diabetes
	Significant co-existent medical condition (e.g. overt diabetes complications, cancer, gut mobility or digestion disorder)
	Significant psychological (e.g. anorexia, Bulimia) and/or mental disorders which undermines informed consent
	Dietary allergies or intolerance for the experimental meals
	Lack of internet access on a computer or tablet at home
	Unable to understand written English and provide informed consent

For peer review only

Data collection stages and procedures

The study will proceed in three stages at the St James's hospital (LTHT) with the dietary intervention and interviews conducted remotely (e.g., participant's home) (**Fig 1**). All pregnant women with pre-existing T1DM or T2DM are scheduled for regular NHS clinical visits every two weeks throughout the pregnancy. Each woman has an assigned diabetes midwife who caseloads her pregnancy and liaises with the rest of the clinical care team. Due to COVID-19 and intermittent lockdowns, pregnant women only attend face-to-face meetings at the clinic when due for a scan (*a dating scan* at 10-12 weeks, an *anomaly scan* at 18-20 weeks and *growth scans* at 26-28, 32-34, and 36 weeks of pregnancy) unless deviated due to complications or early delivery. All women with T1D and T2D are currently offered CGM as part of their clinical care. The CGM data is automatically uploaded to a secure, remote clinical database, where it can be securely accessed and downloaded by the clinical team and authorised researchers.

The study will require patient consent (i) permitting secure access to routinely collected clinical details regarding maternal and offspring health at each clinical visit and delivery (i.e., height, weight, blood pressure, HbA1c, lipids), continuous glucose monitoring (CGM) data, and delivery outcomes with approved study researchers; (ii) permitting researchers to use the residual urine from routine clinically collected samples for metabolite analysis; (iii) to conduct online and interview questionnaires to assess diet and lifestyle during each trimester at ~10-12 weeks, ~18-20 weeks, and ~28-34 weeks; and (iv) a 10ml blood sample to be taken with routine clinical bloods at ~10-12 weeks, ~18-20 weeks, and ~28-34 week visits for subsequent metabolomic and genetic analysis. Each participant will be contacted three times for a phone or video chat (participant preference) for ≤ 30 minutes each within 2 weeks of each clinical appointment.

Patient and Public Involvement: Patients or the public have not been involved in the design of this pilot and feasibility study. However, upon completion of the study, participants will be invited to provide insight and comments regarding the study itself, the burden enrolment and intervention, and identifying other research priorities relevant to the health condition that the researchers can integrate into future studies. They will also be asked if they consent to follow-up discussions and are keen to be updated on study results and publication material. These points will be invaluable for guiding future work in this area.

First call 10-12 weeks of pregnancy: A member of the research team will provide details of the study with the participant and answer any questions. The participants will be instructed to

For peer review only

record their dietary intake for three days, including two weekdays and one weekend, using the myFood24 app. MyFood24 is a validated online food diary system created to analyse nutritional intake which has previously been used in pregnancies complicated by diabetes[18]. Details on participant's recent physical activity levels (Par-Q for pregnancy)[19] and sleep quality (Leeds Sleep Evaluation)[20] will be recorded by interview (**Supplementary Material, F-G**), while patterns and habitual mealtimes will be collected by myFood24[21]. The dietary meal intervention in the study will also be discussed.

Second call between 18-20 weeks of pregnancy: Participant's compliance in the study and details on physical activity, sleep and habitual mealtimes will be re-recorded. A reminder and summary of when and how to use the myFood24 app will be given. The participant will also be instructed about how and when their study intervention's first set of experimental meals should be consumed.

Third call between 28-34 weeks of pregnancy: Participant's compliance and information transfer as in the second call will be repeated. The participant will also be instructed about how and when their study intervention's second set of experimental meals should be consumed. Subsequently, the participant will be thanked for their participation in the study and not contacted again after this point.

Nested cross-over dietary meal intervention within the study

Shortly after their ~18-20 week and ~28-34-week clinical visit, participants will be asked to consume standardised breakfast meal replacements in their own home, at breakfast time for 4 days. These will be two different experimental meals (A and B; matched for 400 kcal and 13 g of fat) consumed under free-living conditions. The experimental meals will appear and taste similar but differ in protein quantity which will alter how quickly the glucose in each meal is absorbed into the blood[22]. One experimental meal will have 20g of vegan and gluten-free protein powder added, which slow gastric emptying and glucose absorption into the blood to a rate that is comparable to commonly consumed whole grain breakfast cereals (e.g., steel-cut or rolled oats; GI≈40). The other experimental meal will have no added protein. The experimental meals (labelled A or B) with a drink shaker in a box by the research team in a COVID-safe lab. Each participant will be assigned to one of six random orders to consume the experimental breakfast meals (AABB, ABAB, BBAA, BABA, ABBA, BAAB) over 4 days using an online randomiser (https://www.random.org/integer-sets/). This will be done by block randomization to assign 12-13 participants to each of the six possible orders of meal consumption, which the

For peer review only - http://bmjopen.bmj.com/site/about/guidelines.xhtml

participant will follow for both sets of meals (i.e., ~18-20 week and ~28-34 wks). The participant will only need to pour the powder into the shaker, add cold water to the line marked on the cup (500 mL), and consume within 5 minutes.

The powder is a nutritionally complete meal replacement in drink form, every meal contains a balance of protein, carbohydrates, essential fats, fibre, plus all 26 essential vitamins and minerals, and phytonutrients. Additionally, the product is low in sugar, lactose-free, contains no nuts or palm oil, and has a long shelf life. The powder is commercially available and is produced in facilities that meet highest quality standards. This product was chosen to minimise time-burden for participants and it is free from many commonly avoided food items (i.e., lactose, nuts, gluten, and meat).

Participants will be asked to avoid consuming other foods and drinks (aside from water) for two hours, after which they may consume food as usual. However, they are freely permitted to measure their own blood glucose levels at any point and will be advised to manage any hyperglycaemic or hypoglycaemic events, even if this means eating or drinking within 2 hours of the meal. Participants will be asked to inform the research team of any events by email as soon as possible.

Data management

The research team will assign unique random screening IDs at the recruitment phase. The study ID will be used to pseudonymise (using personal participant identification numbers; PID) and harmonise the data shared by the NHS clinical database (clinical records) with the data collected by the research team at the University of Leeds (i.e., questionnaires, metabolite, and genetic data). Only the clinical team and authorised members of the research team will be able to link the study ID to the participant. All personally identifiable information will be stored in a password protected and encrypted database in a secure area.

Clinical data: Standard care to measure and collect maternal anthropometric, glycaemic, medication, lipid levels, and blood pressure information during routine hospital visits and during and after labour. Offspring anthropometry measures are taken by the direct healthcare team, which is part of routine care. The women will be asked to give consent for the research team to access their clinical records to obtain this data.

CGM: Standard clinical care in T1D and T2D pregnancies, women will be asked to give consent for the research team to access their CGM data. Of the numerous metrics provided by

For peer review only

CGM – i.e., area under the curve (AUC), mean glucose, coefficient of variation (CV) of glucose, mean amplitude of glucose excursions (MAGE), and time in range (TIR) – AUC will be the primary metric for the study.

Urine samples: Standard clinical care, we will ask for up to 2.5 mL of any urine not required for clinical analysis to be saved for research use. These samples will be stored for subsequent metabolic analysis. All samples will be stored at the University of Leeds in designated Human Tissue Act approved and compliant facilities.

Blood samples: Standard clinical care requires blood samples for analysis. At the time of routine collection at each clinical visit, an additional 10 ml blood will be collected for this study. These blood samples will be stored for subsequent metabolic and genomic analysis (relevant to nutrition/diabetes/pregnancy and fetal growth). All samples will be stored at the University of Leeds in designated and secure facilities.

Lifestyle questionnaires: At three time points across pregnancy (~10-12, ~18-20, and ~28-34 weeks gestation), a designated member of the research team will call (phone or video) the participant to complete the questionnaires on physical activity, sleep quality/patterns, and habitual mealtime habits.

Dietary records: At two timepoints across pregnancy (~18-20 and ~28-32 weeks of gestation), each participant will be asked to record their diet for 3 days (2 week days, 1 weekend day) using a validated online semi-quantitative food frequency questionnaire (myFood24), which estimates dietary intake data (i.e., macronutrients, micronutrients and vitamins for up to 220 nutrients) according to McCance and Widdowson (7th edition) and branded items that offer nutritional data[23]. Briefly, the data is provided to researchers as a spreadsheet with anonymised identifiers for each participant that can be directly imported into R for analysis. The performance of myFood24 and telephone-based 24 hour dietary recall are in agreement (interclass correlation 0.4-0.5).[23]

Outcomes of interest: The primary outcome of interest is CGM glucose data, with AUC glucose as the primary CGM metric of interest. Secondary outcomes of interest are associations between metrics of dysglycemia during pregnancy with maternal outcomes (e.g. gestational weight gain, pre-eclampsia, hypertension, mode of birth, birth trauma, pre-term delivery, and metabolism), and infant outcomes (e.g. birth weight, height, pre-term delivery, mortality, birth trauma, hypoglycaemia, congenital malformation, head and abdominal circumference,

For peer review only

perinatal morbidity), and the moderating effects of genetics, metabolism, and diet and lifestyle. All secondary analyses are considered exploratory.

Statistical analysis considerations

All standard CGM metrics will be calculated, with AUC glucose as the primary CGM metric (mean±SD). The primary analysis will be the effect of dietary protein on AUC glucose for the 24-hr window immediately after each study meal. The analysis will be constructed as pairwise linear model with study meal (0 = low protein, 1 = high protein) regressed against 24-hr mean AUC glucose and adjusted for study parameters (e.g., randomised meal order) and participant covariates (e.g., maternal age, ethnicity, parity, BMI, gestational age, physical activity, and sleep quality). Statistical significance will be set at $p<0.05$, where a $p<0.05$ for study meal will suggest a significant effect of dietary protein on 24-hr postprandial AUC glucose. The direction and significance of covariates will be investigated to identify study and participant mediators of the association. Statistical analysis will be conducted in R studio and SPSS.

Secondary research objectives include (i) the association between dysglycemia and maternal and newborn health, and (ii) the association between maternal diet in early pregnancy with dysglycemia during pregnancy. These analyses will also be performed using regression models adjusted for covariates, with the assessment of early diet on longitudinal changes in dysglycemia also adjusted for timepoints of AUC (i.e., mixed-model). The association between early maternal diet and AUC, will be performed using three distinct dietary metrics (calculated from myfood24):

(1) *Overall diet quality*: The association between diet quality and dysglycemia will be assessed using an overall diet quality score[24]. This scoring method has been modified and used previously to assess maternal diet quality in a multi-ethnic prospective birth cohort[25]. The modified Alternative Healthy Eating Index (mAHEI) score is calculated using the following method; an individual will receive 10 points for each of the following food categories when they consume above or below a threshold of: ≥5 servings vegetables, ≥4 servings fruit, ≥1 serving nuts or soy proteins, ≥3 servings whole grains, a ratio of ≥4 servings fish to 1 serving meat and eggs, and ≤0.5 servings of less-healthy foods (i.e., fried foods and processed meats). Intermediate intakes are scored proportionally between 0 and 10. The maximum mAHEI score is 60; the higher the score the more healthful the participant's diet.

For peer review only

(2) *Macronutrient*: Daily macronutrient consumption (total carbohydrates, proteins, and fats) and markers of GI quality (fibre, sugars) will be adjusted for energy and regressed against CGM measures of dysglycemia. Doing so, will allow for the contribution of individual macronutrients on glycemic measures to be evaluated.

(3) *Cardinal Foods*: Partial-least Squares (PLS) will be used to identify foods that are more commonly observed in participants with favourable or unfavourable glycemic control (identified above/below median for glycemic measures). These foods will then be investigated for their association with measures of dysglycemia using a regression model.

Quality control: All participants will receive standard clinical care as per NICE guidance, which will minimise researcher bias. The primary outcome measures are based on laboratory measurements and predetermined cut-off values, which the researchers will not be able to influence. We do not foresee significant researcher bias in collecting antenatal and perinatal outcome data because these will be obtained by clinical staff who are independent of the study outcome and from the participants' medical records. We do not foresee any significant researcher bias in collecting lifestyle records, as standardised and validated questionnaires will be used to obtain this information. Furthermore, all participants will be asked to consume the two standard meals but the order of their consumption will be randomised. We do not foresee any conflict of interests. The data collected will not be used for informing clinical care decisions of specific cases, all the women will continue with their usual clinical care pathway for the duration of their pregnancy, and all women will be free to terminate their participation in the study at any time with no effect on their quality of care.

For peer review only

ETHICS & DISEMMINATION

Ethics approval: This study has been reviewed and approved by the Leeds East Research Ethics Committee at the University of Leeds (21/NE/0196).

Dissemination: There is no formal interim analysis planned except the ongoing evaluation of the recruitment numbers. Results will be disseminated in peer reviewed scientific journals, conference presentations, and publication on (social) media and in newsletters, to inform the participants and wider public.

DISCUSSION

The INFORMED clinical trial is double-blinded cross-over randomised clinical trial to evaluate the effect of dietary protein within experimental meals on dysglycemia in women with pre-existing type-1 or type-2 diabetes. Additionally, we will explore (i) the association between dysglycemia and maternal and newborn health, and (ii) the association between maternal metabolic profiles in early pregnancy with dysglycemia in later pregnancy, which may provide insights into novel precision therapies for women with DIP[26]. The identification of dietary mediators of glucose variability will aid in the development of more efficacious and appropriate strategies to control glucose levels and minimise maternal and offspring risks in women with DIP[12 13 26].

AUTHOR CONTRIBUTIONS: Cassy Dingena: Conception, original draft preparation, writing - review & editing, approved the final manuscript. **Anvesha Mahendra**: Original draft preparation, writing - review & editing, approved the final manuscript. **Melvin Holmes**: Supervision, Writing - review & editing, approved the final manuscript. **Naomi S Clement**: Writing - review & editing, approved the final manuscript. **Eleanor Scott**: Conception, supervision, writing - review & editing, approved the final manuscript. **Michael A Zulyniak**: Conception, supervision, Original draft preparation, writing - review & editing, approved the final manuscript.

ACKNOWLEDGEMENTS: myFood24 was developed through Medical Research Council funding, grant G110235. myfood24 is now being supported by spinout company Dietary Assessment Ltd. Requests to use myfood24 should be made to enquiries@myfood24.org.

FUNDING STATEMENT: This work was supported by Wellcome Trust UK (MAZ; 217446/Z/19/Z) and the University of Leeds (CD; N/A).

COMPETING INTEREST STATEMENT: No competing interest.

REFERENCES:

1. Macintosh MCM, Fleming KM, Bailey JA, et al. Perinatal mortality and congenital anomalies in babies of women with type 1 or type 2 diabetes in England, Wales, and Northern Ireland: population based study. *Bmj* 2006;333(7560):177.

For peer review only

2. Murphy HR, Howgate C, O'Keefe J, et al. Characteristics and outcomes of pregnant women with type 1 or type 2 diabetes: a 5-year national population-based cohort study. *The lancet Diabetes & endocrinology* 2021;9(3):153-64.
3. Symonds ME, Ramsay MM. Maternal-fetal nutrition during pregnancy and lactation: Cambridge University Press 2010.
4. Lapolla A, Metzger BE. Gestational Diabetes: A Decade After the HAPO Study: Karger Medical and Scientific Publishers 2019.
5. Federation ID. International Diabetes Federation. IDF Diabetes Atlas, 9th edn. Brussels, Belgium: 2019, 2019.
6. Tennant PWG, Glinianaia SV, Bilous RW, et al. Pre-existing diabetes, maternal glycated haemoglobin, and the risks of fetal and infant death: a population-based study. *Diabetologia* 2014;57(2):285-94.
7. National Institute for Clinical E. Diabetes in pregnancy: management from preconception to the postnatal period. *NICE Guidelines [NG3] Published February* 2015
8. Smith R, Ridout A, Livingstone A, et al. Motivational interviewing to increase physical activity in women with gestational diabetes. *British Journal of Midwifery* 2021;29(10):550-56.
9. American Diabetes A. Management of diabetes in pregnancy. *Obstetrical & Gynecological Survey* 2017;72(5):264-66.
10. Dingena CF, Holmes MJ, Campbell MD, et al. Observational assessments of the relationship of dietary and pharmacological treatment on continuous measures of dysglycemia over 24 hours in women with gestational diabetes. *medRxiv* 2022:2022.09.16.22280027. doi: 10.1101/2022.09.16.22280027
11. Yu Q, Aris IM, Tan KH, et al. Application and utility of continuous glucose monitoring in pregnancy: a systematic review. *Frontiers in Endocrinology* 2019:697.
12. Perichart-Perera O, Balas-Nakash M, Rodríguez-Cano A, et al. Low glycemic index carbohydrates versus all types of carbohydrates for treating diabetes in pregnancy: a randomized clinical trial to evaluate the effect of glycemic control. *International journal of endocrinology* 2012;2012
13. Moses RG, Luebcke M, Davis WS, et al. Effect of a low-glycemic-index diet during pregnancy on obstetric outcomes. *The American Journal of Clinical Nutrition* 2006;84(4):807-12.
14. Scott EM, Murphy HR, Kristensen KH, et al. Continuous Glucose Monitoring Metrics and Birth Weight: Informing Management of Type 1 Diabetes Throughout Pregnancy. *Diabetes Care* 2022;45(8):1724-34. doi: 10.2337/dc22-0078
15. Fabricatore AN, Ebbeling CB, Wadden TA, et al. Continuous glucose monitoring to assess the ecologic validity of dietary glycemic index and glycemic load. *The American journal of clinical nutrition* 2011;94(6):1519-24.
16. Law GR, Alnaji A, Alrefaii L, et al. Suboptimal nocturnal glucose control is associated with large for gestational age in treated gestational diabetes mellitus. *Diabetes care* 2019;42(5):810-15.
17. Faul F, Erdfelder E, Buchner A, et al. Statistical power analyses using G*Power 3.1: Tests for correlation and regression analyses. *Behavior Research Methods* 2009;41(4):1149-60. doi: 10.3758/BRM.41.4.1149
18. Morris MA, Hutchinson J, Gianfrancesco C, et al. Relationship of the frequency, distribution, and content of meals/snacks to glycaemic control in gestational diabetes: the myfood24 GDM pilot study. *Nutrients* 2019;12(1):3.
19. Charlesworth S, Foulds HJA, Burr JF, et al. Evidence-based risk assessment and recommendations for physical activity clearance: pregnancy 1 This paper is one of a selection of papers published in this Special Issue, entitled Evidence-based risk

For peer review only

assessment and recommendations for physical activity clearance, and has undergone the Journal's usual peer review process. *Applied Physiology, Nutrition, and Metabolism* 2011;36(S1):S33-S48. doi: 10.1139/h11-061 %M 21800947
20. Parrott AC, Hindmarch I. The leeds sleep evaluation questionnaire in psychopharmacological investigations—a review. *Psychopharmacology* 1980;71(2):173-79. doi: 10.1007/BF00434408
21. Gianfrancesco C, Darwin Z, McGowan L, et al. Exploring the Feasibility of Use of An Online Dietary Assessment Tool (myfood24) in Women with Gestational Diabetes. *Nutrients* 2018;10(9) doi: 10.3390/nu10091147 [published Online First: 2018/08/26]
22. Meng H, Matthan NR, Ausman LM, et al. Effect of macronutrients and fiber on postprandial glycemic responses and meal glycemic index and glycemic load value determinations. *The American journal of clinical nutrition* 2017;105(4):842-53.
23. Wark PA, Hardie LJ, Frost GS, et al. Validity of an online 24-h recall tool (myfood24) for dietary assessment in population studies: comparison with biomarkers and standard interviews. *BMC medicine* 2018;16(1):136. doi: 10.1186/s12916-018-1113-8
24. Dehghan M, del Cerro S, Zhang X, et al. Validation of a semi-quantitative Food Frequency Questionnaire for Argentinean adults. *PLoS One* 2012;7(5):e37958.
25. NutriGen Alliance I, de Souza RJ, Zulyniak MA, et al. Harmonization of food-frequency questionnaires and dietary pattern analysis in 4 ethnically diverse birth cohorts. *The Journal of nutrition* 2016;146(11):2343-50.
26. Schaefer-Graf U, Napoli A, Nolan CJ. Diabetes in pregnancy: a new decade of challenges ahead. *Diabetologia* 2018;61(5):1012-21.

FIGURE LEGENDS

Figure 1. Study Flowchart. Participant will be monitored and in contact with the clicnial and research team through the sutdy, starting at 10-12 weeks gestation. At each clinical visit, routine data will be collected from each particpant as stadard of care (e.g., anthropometrics, blood samples and CGM). This informaiton will be supplemented with a lifestyle information (e.g., diet and sleep) collected directly from the particiapnt via electronic and internet questionaires. The maternal and offspring data available for analysis at each timepoint is listed below the timeline. The interventions will be delivered at two timepoints (18-20 weeks and 34-36 weeks). CGM: continuous glucose monitoring

For peer review only

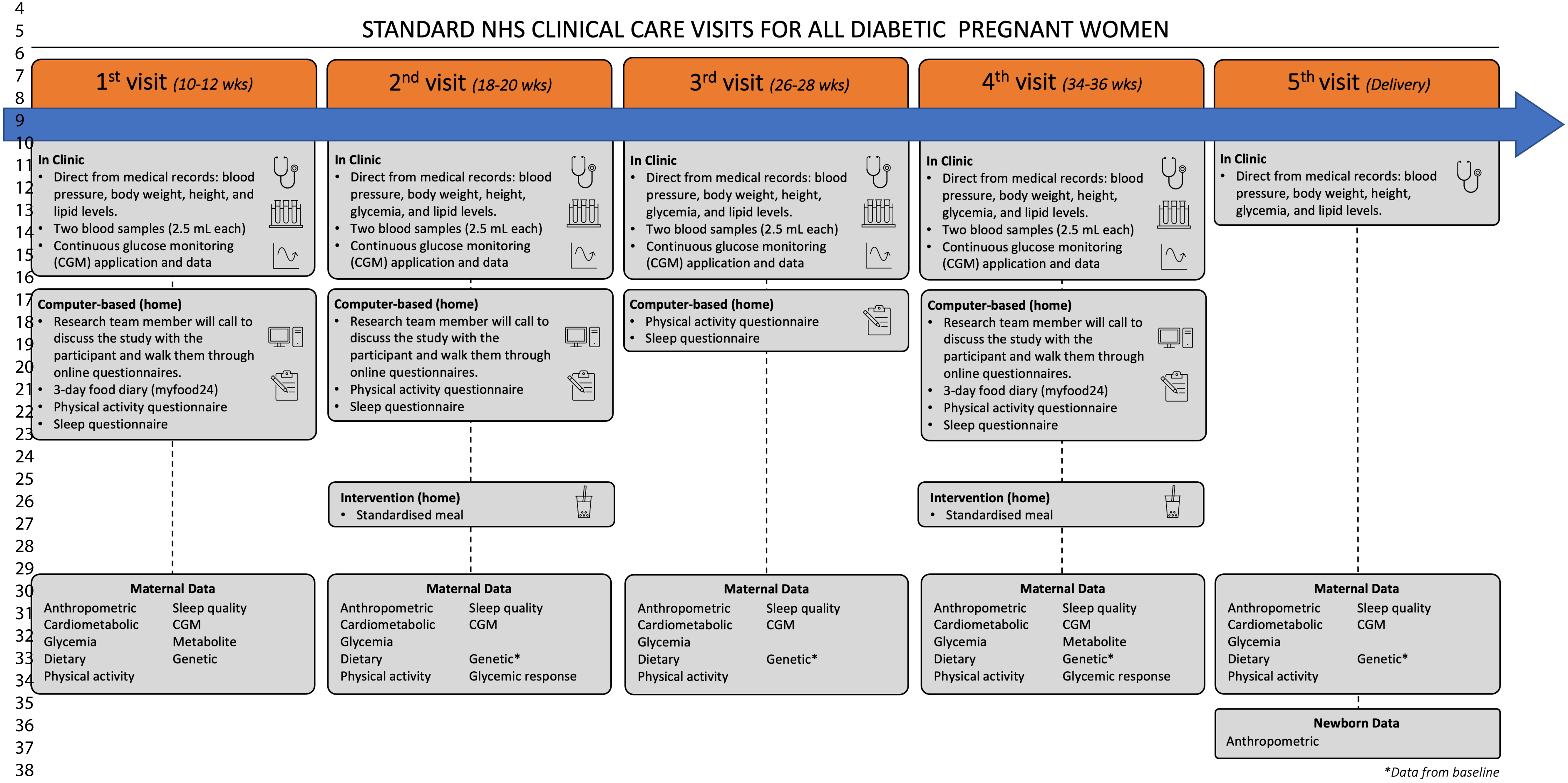
STANDARD NHS CLINICAL CARE VISITS FOR ALL DIABETIC PREGNANT WOMEN
1st visit (10-12 wks)
2nd visit (18-20 wks)
3rd visit (26-28 wks)
4th visit (34-36 wks)
5th visit (Delivery)
In Clinic
Direct from medical records: blood pressure, body weight, height, and lipid levels.
Two blood samples (2.5 mL each)
Continuous glucose monitoring (CGM) application and data
Computer-based (home)
Research team member will call to discuss the study with the participant and walk them through online questionnaires.
3-day food diary (myfood24)
Physical activity questionnaire
Sleep questionnaire
Maternal Data
Anthropometric
Cardiometabolic
Glycemia
Dietary
Physical activity
Sleep quality
CGM
Metabolite
Genetic
In Clinic
Direct from medical records: blood pressure, body weight, height, glycemia, and lipid levels.
Two blood samples (2.5 mL each)
Continuous glucose monitoring (CGM) application and data
Computer-based (home)
Research team member will call to discuss the study with the participant and walk them through online questionnaires.
Physical activity questionnaire
Sleep questionnaire
Intervention (home)
Standardised meal
Maternal Data
Anthropometric
Cardiometabolic
Glycemia
Dietary
Physical activity
Sleep quality
CGM
Genetic*
Glycemic response
In Clinic
Direct from medical records: blood pressure, body weight, height, glycemia, and lipid levels.
Two blood samples (2.5 mL each)
Continuous glucose monitoring (CGM) application and data
Computer-based (home)
Physical activity questionnaire
Sleep questionnaire
Maternal Data
Anthropometric
Cardiometabolic
Glycemia
Dietary
Physical activity
Sleep quality
CGM
Genetic*
In Clinic
Direct from medical records: blood pressure, body weight, height, glycemia, and lipid levels.
Two blood samples (2.5 mL each)
Continuous glucose monitoring (CGM) application and data
Computer-based (home)
Research team member will call to discuss the study with the participant and walk them through online questionnaires.
3-day food diary (myfood24)
Physical activity questionnaire
Sleep questionnaire
Intervention (home)
Standardised meal
Maternal Data
Anthropometric
Cardiometabolic
Glycemia
Dietary
Physical activity
Sleep quality
CGM
Metabolite
Genetic*
Glycemic response
In Clinic
Direct from medical records: blood pressure, body weight, height, glycemia, and lipid levels.
Maternal Data
Anthropometric
Cardiometabolic
Glycemia
Dietary
Physical activity
Sleep quality
CGM
Genetic*
Newborn Data
Anthropometric
*Data from baseline

UNIVERSITY OF LEEDS

The Leeds Teaching Hospitals NHS Trust

INFORMED

Individualised patieNt care and treatment FOR MatErnal Diabetes

SUPPLEMENTARY MATERIALS

Supplementary section: A

INFORMED

INDIVIDUALISED PATIENT CARE AND TREATMENT FOR MATERNAL DIABETES

UNIVERSITY OF LEEDS

The Leeds Teaching Hospitals NHS Trust

THE MAIN ISSUE?

A mother's blood **glucose changes** after mealtimes and throughout the day, affected by her **personal characteristics**, daily lifestyle and the pregnancy itself. **Too much or uncontrolled glucose** in your blood during pregnancy can lead to a large baby and can cause problems during pregnancy and labour. Also, babies exposed to higher glucose levels are more likely to become **obese** and get **Type 2 diabetes** when they grow up.

WHAT WILL WE INVESTIGATE?

Recent studies have shown that **other factors beyond the characteristics of food** play an important role in how glucose is absorbed after a meal during pregnancy. These factors include your **personal charactristics** such as age, ethnicity and BMI and genetics.

Using **continuous glucose monitoring** (CGM), which measures **glucose levels every few minutes**, we will investigate:

1) How **diet quality effects glucose control** in type 1 and 2 diabetes pregnancy?
2) Which **personal characteristics** are most strongly related?
3) How does **glucose control** evolve during pregnancy?

INTERESTED?

If you have **type 1 or type 2 diabetes** and are **within the first 12 weeks** of pregnancy and interested in taking part, please contact us for more information.

+31627072821

fscd@leeds.ac.uk

WHAT WE WILL ASK FROM YOU?

During your routine care, medical details are recorded and you will wear a CGM device. We ask for your permission to safely access and assess this data.

Furthermore, we ask you to complete short questionnaires on diet, physical activity and sleep at three occasions during your pregnancy (after each routine care visit). These questionnaires will be partly online and via phone calls.

To gain more insight in mealtime glucose responses, we ask you to consume standardised breakfast meals on two separate occasions (optional), no addtional clinical visits needed. These meals will be delivered at your home.

HOW LONG DOES THE STUDY LAST?

We will collect data throughout the pregnancy, inluding birth outcomes.

WHAT WILL THIS TELL US?

We think that all the information we gather will help to develop new ways in which women can reduce their risk of having uncontrolld glucose, reduce still births, pregnancy complications and improve the long term health of their children.

INFORMED Infographic (version 1.0) IRAS No: 297276 Date: 02-07-2021

For peer review only - http://bmjopen.bmj.com/site/about/guidelines.xhtml

UNIVERSITY OF LEEDS

The Leeds Teaching Hospitals NHS Trust

Supplementary section B: Participant Information Sheet

INFORMED

Individualised patieNt care and treatment FOR MatErnal Diabetes

Understanding the glycaemic profile of maternal diabetes using continuous glucose monitoring: intensive glucose profiling to inform patient care and treatment

Participant Information Sheet

Thank you for considering your participation in our study called INFORMED, which is part of a PhD research project at the School of Food Science and Nutrition. We - as research team - would like to provide you with details about the study, what your role will involve, and other key information before you decide.

Please ask us (*contact details at the end of the handout*) if there is anything that is not clear or you would like more information. Take time to decide whether or not you wish to take part.

Study information

What is the purpose of the study?

During pregnancy, a mother's blood glucose level changes constantly across 24-hour period, and is affected by her physical characteristics, lifestyle, and the pregnancy itself. While many factors affect the way babies grow in the womb, one of the easiest to measure and modify, is the amount of glucose that they get from their mother. Uncontrolled or too much glucose in their mother's blood during pregnancy, usually leads to a large baby and can increase the chance of problems during pregnancy, labour, and immediately after birth for both mother and child. Being born too small can also be problematic and has been linked to increases the chances of obesity and type 2 diabetes.

Glucose levels of the mother rise after meal consumption and, if uncontrolled, can contribute to some of these health concerns. While the type of food being eaten is vital, recent studies have shown that other factors (such as age, ethnicity, activity levels, and sleep duration) also play a part. However, despite knowing these factors, we currently do not know how to modify a meal to match a mother's characteristics and how a mother's diet effects glucose levels throughout pregnancy. As part of your routine care you are wearing a continuous glucose monitor. With this study we are investigating the impact of diet and lifestyle affects on glucose control throughout pregnancy as there is currently very little information on how diet and lifestyle affects glucose levels measures during pregnancy.

Therefore, as a first step, we want to monitor and study how 24-hour and mealtime glucose levels change in response to diet and across pregnancy in women with pre-existing type 1 or type 2 diabetes. Most information on glucose control and medical data will be requested via your medical records, if you give us permission for us to access your data. To decrease the burden of participation, we will use existing data as much as possible. However, to be able assess your diet and lifestyle during pregnancy, we will ask you to complete questionnaires via phone calls. These questionnaires are detailed below. None of the information obtained via the questionnaires will be shared with your clinical care team. This data on lifestyle will be anonymised and is solely for the purpose of this study.

For peer review only - http://bmjopen.bmj.com/site/about/guidelines.xhtml

UNIVERSITY OF LEEDS

The Leeds Teaching Hospitals NHS Trust

Why have I been invited?

We are approaching women with type 1 and type 2 diabetes, who are early in their pregnancy, to help us with this study. You do not have to take part – it is completely up to you — and does not affect the quality of care you receive from the NHS. Also, if you choose to take part and later decide to withdraw (for any or no reason), it will not affect your quality of care from the NHS.

What could my participation do?

By taking part in this study, you will help us to better understand how glucose levels change during pregnancy in women with type-1 and type-2 diabetes and the role of diet. By knowing this, we can design special diets and nutritional strategies to minimise the chances of babies being exposed to abnormal glucose levels and their risk of future health problems.

What would taking part involve?

Medical information. We ask you to give us consent to access selected parts of your medical record that reflect your general health and the health of your pregnancy (e.g., blood pressure, blood/urine test results, current medication, diabetes related pregnancy outcomes) and your diabetes health risks (e.g., age, body weight, ethnicity). Additionally, once you have given birth, your baby's birthweight, and any pregnancy complications will be copied from your medical records.

Urine samples. You will be providing urine samples regularly during pregnancy to your clinical team. Once they have been tested, rather than throwing them away we would like your consent to keep the remaining sample for future metabolic analysis.

Blood Samples. You will be having blood taken regularly during this pregnancy for your clinical care. On three of your routine visits to the Diabetes in Pregnancy Clinic we would like your consent to take an additional 10ml of blood for the study. We will store this to look at molecular and genetic markers that may be involved in metabolism and diabetes later. No infant blood samples are requested.

All samples will be stored at the University of Leeds in designated Human Tissue Act approved and compliant facilities.

Glucose Data. We ask for your consent for us to access your clinical glucose data throughout pregnancy. This will require no additional work on your part.

Lifestyle Questionnaires. On three occasions during your pregnancy, at ~10-12, ~18-20, and ~28-34, we will contact you at your convenience by phone or video call to complete some short questionnaires about your habitual physical activity, sleep quality / patterns and mealtimes. Also, following these three clinical visits, we will ask you to keep track of your diet for 3 days (2 weekdays and 1 weekend day) using an online dietary tracker called MyFood24. During the phone call, we will explain you how to use this dietary tracker. The phone calls will last not more than 30 minutes. The dietary tracker will take approximately 10 minutes per day to complete. This data will be anonymised and none of this data will be shared with your clinical care team.

Breakfast replacements. To gain more insight into mealtime glucose responses, we would like you to consider taking part in additional part of the study, where we will provide you with two different breakfast shakes to drink instead of your usual breakfast for 4 days on two separate occasions (one during your 2nd and one during your 3rd trimester). The two different breakfast shakes (e.g. Shake 1 and Shake 2) have the same amount of carbohydrate as that recommended during pregnancy, but one is designed to be absorbed slower, and the other faster so we can see how this affects your glucose measures on the continuous glucose monitor. Dependent on your randomization you will consume Shake 1 for two days followed by Shake 2 or vice versa. They are vegan friendly. The shakes will be delivered to your home with instructions for you to prepare.

For peer review only - http://bmjopen.bmj.com/site/about/guidelines.xhtml

You can of course still participate in the main study without having to take the breakfast shakes if you prefer.

This study will tell us, in detail previously unseen, how (mealtime) glucose changes across pregnancy and how diet can best be used to manage glucose levels and minimise maternal and infant health risks.

What are the possible risks of taking part?
Although we have designed the meals to not contain allergens and to release the same amount of glucose as you would usually eat for breakfast there is a possibility that you may experience an allergic reaction or higher glucose levels than normal after the standardised meal consumption. We will check that you have no allergies before taking part and ask you to contact the research team if you have these reactions to the meal. You will be advised to monitor and manage your blood glucose levels like you normally would and feel most comfortable with. However, if blood glucose levels surpass >18mmol/L for more than 90-minutes you are advised to administer a corrective dose of insulin or contact your GP/clinical care team. The meals are designed to minimize risk of hyperglycaemia. Blood samples are part of your routine clinical care and will be performed by qualified clinical staff, so any discomfort should be minimal.

What are the possible benefits of taking part?
There are no specific benefits to you of taking part, but participating in this study will give us important information about how to assess glucose in relation to personal characteristics, pregnancy outcomes, and newborn health. We anticipate that this will then help us to identify and develop new diet strategies to help women reduce their risk of small or large babies, stillbirths, pregnancy complications, and improve the long-term health of their children.

Further Information

What will happen if I don't want to carry on with the study?
You are free to withdraw at any time without explanation. If you decide not to carry on, it will not affect your care in anyway.

What if something goes wrong?
During the study, you will be covered by the Sponsor's Insurance, the University of Leeds is acting as Sponsor for this study. The University of Leeds has insurance cover in force, which meets claims against it and where those claims arise from the Universities own negligence in its role and activities relating to the study (and which is subject to the terms, conditions and exceptions of the relevant policy). Clinical negligence indemnification will rest with the participating NHS Trust under standard NHS arrangements.

If you are unhappy about any part of the study, you are encouraged to discuss this with the research team or with the Patient Assistance and Liaison Services (PALS) at your hospital. Normal legal processes are also open to you. We foresee minimal risks as most data will be collected from your routine clinical records and specific study risks are limited to questionnaires and meal replacements.

What will happen to my additional blood samples and urine sample?
Your blood and urine samples will be labelled with your unique study number and stored in freezers at the University of Leeds for longer term storage. Analysis of the samples will be undertaken for molecular and genetic factors that may contribute to a mother's metabolism, glucose control and babies growth. Only researchers directly involved in the study will have access to the samples. Results of samples analyses will only be used for the purpose of the research study.

How will we use information about you?
We will need to use information from you and from your medical records (including the continuous glucose monitoring data) for this research project. This information will include your initials/NHS number/date of birth/name/contact details. A member of your clinical care team will give you a unique random study

For peer review only - http://bmjopen.bmj.com/site/about/guidelines.xhtml

number, so that personal information cannot be traced back by members of the research team. Your consented medical data will be routinely stored on an electronic patient platform using your study number. Only authorised members of the research team will be able to access this platform and copy the data to secure university computers, this data will be stored for a maximum of 15 years, for analysis and writing up the results and also for authorised people to check your records to make sure that the research is being done properly. These people who do not need to know who you are will not be able to see your name or contact details. Your data will have a unique study number that will make you anonymous to the study team. We will keep all information about you safe and secure. Once we have finished the study, we will keep some of the data so we can check the results. All analyses and reports will be written in a way that no-one can work out that you took part in the study.

How will my information be kept confidential?
All information which is collected about you will be held securely and treated in accordance with the Regulation (EU) 2016/679 (General Data Protection Regulation) and the Data Protection Act 2018.

We will be using information collected by your local hospital from you and your medical records in order to undertake this study. No personal identifiable data will leave the NHS hospital without your consent; Data leaving the hospital will be labelled with your unique study number and will not have your name or any other identifying details on it. We refer to this as linked anonymised data as it is linked to you by a code. The code will only be known by key research team members. It will be kept securely.

Data which leaves the NHS Trust where you are being treated will be held securely in a database, operated by the data analysis team at the University of Leeds. This includes only linked anonymised study data and will not have your name or any other identifying details on it.

If you join the study, the data collected for the study, together with any relevant medical records, may be looked at by authorised persons from University of Leeds, the Research and Development Department of your local hospital and the Regulatory authorities to check the study is being carried out correctly. They all have a duty of confidentiality to you as a research participant.

Other third party researchers (e.g. universities, NHS organisations or companies involved in health and care research) may wish to access anonymised data (including samples) from this study in the future (anonymised data do not include names, addresses, or dates of birth, and it is not possible to identify individual participants from anonymised data). If this is the case, the Chief Investigator will ensure that the other researchers comply with legal, data protection and ethical guidelines. This may include research outside of the UK and EU and/or research that is commercial in nature. Your data will be stored securely for a period of 15 years after the end of the trial before being destroyed.

What are your choices about how your information is used?
If you withdraw consent during the study, no further data will be collected on you. However, any data (including samples) already collected by the research team may be retained and subsequently analysed for the purposes of the study. Your right to access, change or move your information are limited, as we need to manage your information in specific ways in order for the research to be reliable and accurate. To safeguard your rights we will use the minimum personally-identifiable information possible.

The University of Leeds as the Sponsor, is the data controller for this study. This means that we are responsible for looking after your information and using it properly.

The University of Leeds is the data processors for this study. The lawful basis for processing personal data collected in this study is that it is a task in the public interest. You can find out more about how we use your information at https://dataprotection.leeds.ac.uk/wp-content/uploads/sites/48/2019/02/Research-Privacy-Notice.pdf; and https://dataprotection.leeds.ac.uk/wp-content/uploads/sites/48/2019/09/HRA-

For peer review only - http://bmjopen.bmj.com/site/about/guidelines.xhtml

UNIVERSITY OF LEEDS

The Leeds Teaching Hospitals NHS Trust

transparency-wording.pdf by contacting University of Leeds Data Protection Officer's (e-mail: dpo@leeds.ac.uk).

What will happen to the study results?

The study is part of a PhD project and the result will be used for writing the doctorate thesis. The study results may be presented at meetings or published in scientific journals but individuals will not be identifiable. After the study has ended we will send a newsletter with the study results to your research team, which they will be able to share with you.

Who is organising and funding the research?

The research study has been primarily funded and sponsored by the University of Leeds with additional support from the Wellcome Trust (MZ). The Chief Investigators (Dr Michael Zulyniak and Professor Eleanor Scott) are University of Leeds researchers. Professor Scott is also one of the senior NHS consultants providing clinical care in the Diabetes Pregnancy Clinic.

Who has reviewed the study?

Before any research goes ahead it has to be checked by an Ethics Committee. This study has been reviewed by the Leeds East Research Ethics Committee.

What happens now if I agree to do the study?

The study procedures will be explained to you in more detail by the research team. You will be able to ask questions and voice any queries. If you agree to take part we will ask you to sign a consent form and complete screening questionnaires online to confirm eligibility, this will take approximately 10 minutes. If you are not eligible to participate, information provided prior to participation will be destroyed. The research team will then co-ordinate with you the dates for completing the lifestyle questionnaires, food dairy, and consuming the breakfast replacements

Please, contact the research team for more information:

Co-Investigator

Name: Cassy Dingena
Address: EC Stoner Building, University of Leeds, Woodhouse Ln, LS2 9JT
Email: fscd@leeds.ac.uk
Telephone: +316 27072821

Chief Investigators

Name: Dr Michael Zulyniak
Address: EC Stoner Building, University of Leeds, Woodhouse Ln, LS2 9JT
Email: m.a.zulyniak@leeds.ac.uk
Telephone: +44 (0)113 343 0685

Name: Professor Eleanor Scott
Address: Manny Cussins Diabetes Centre, St James's University Hospital, Leeds, LS9 7TF
Email: eleanor.scott9@nhs.net
Telephone 0113 2065014

For peer review only - http://bmjopen.bmj.com/site/about/guidelines.xhtml

UNIVERSITY OF LEEDS

The Leeds Teaching Hospitals NHS Trust

Supplementary section C: Consent form

INFORMED

Individualised patieNt care and treatment FOR MatErnal Diabetes

Understanding the glycaemic profile of maternal diabetes using continuous glucose monitoring: intensive glucose profiling to inform patient care and treatment

IRAS Project ID: 297276

Participant ID for this study:

Name of Researcher:

CONSENT FORM

Please **initial** all boxes that apply:

1. I confirm that I have read and understand the Participant Information Sheet dated ……………………….. (Version……….) for the above study. I have had the opportunity to consider the information, ask questions and have had these answered satisfactorily. ☐

2. I understand that my participation is voluntary and that I am free to withdraw at any time without giving any reason, without my medical care or legal rights being affected in any way. ☐

3. I understand that relevant sections of my medical records of me and my baby, after their birth until either of us is discharged from hospital and data collected during this study may be looked at by members of the research team, from regulatory authorities or from the NHS Trust / sponsor, in this case the University of Leeds, where it is relevant to taking part in this research. I give permission for these individuals to have access to my records. ☐

4. I understand that my name will not be linked with the research materials, and I will not be identified or identifiable in the report or reports that result from the research. ☐

5. I agree for any unused urine (up to 2.5 mL) collected as routine care during this study to be stored securely long-term at University of Leeds for later analysis for research purposes only. These are considered a 'gift' from me, and may be used in relevant future research (in an anonymised form). I understand that this may involve co-operation with researchers outside of the UK) (*optional*). ☐

For peer review only - http://bmjopen.bmj.com/site/about/guidelines.xhtml

UNIVERSITY OF LEEDS

NHS
The Leeds
Teaching Hospitals
NHS Trust

6. I agree for any additional blood samples collected during this study to be stored securely long-term at University of Leeds for later analysis for research purposes only. These are considered a 'gift' from me, and may be used in relevant future research (in an anonymised form). I understand that this may involve co-operation with researchers outside of the UK) (*optional*). ☐

    a. I give my consent for the taking of an additional blood sample for molecular analysis (*optional*). ☐

    b. I give my consent for the taking of an additional blood sample for genetic/DNA analysis (*optional*). ☐

7. I understand that the information collected about me and my baby may be used to support other ethically approved research in the future, and may be shared anonymously with other researchers. This may include research outside of the UK and EU and/or research that is commercial in nature. ☐

8. I agree for my GP to be informed of my participation in this study. ☐

9. I give consent to the research team to keep my contact details for them to contact me during and after the study (*optional*). ☐

10. I am happy to be contacted about longer term follow up after ending of the study of myself or my baby (*optional*). ☐

11. I agree to take part in the standardised meals study (*optional*). ☐

12. I agree to take part in the study to the sections I have consented to. ☐

Name of participant	Date	Signature

Name of person taking consent	Date*	Signature*

*To be signed and dated in the presence of the participant.

For peer review only - http://bmjopen.bmj.com/site/about/guidelines.xhtml

UNIVERSITY OF LEEDS

NHS The Leeds Teaching Hospitals NHS Trust

Suplementary section D: Screening Questionnaire

INFORMED

Individualised patieNt care and treatment FOR MatErnal Diabetes

Understanding the glycaemic profile of maternal diabetes using continuous glucose monitoring: intensive glucose profiling to inform patient care and treatment

Screening questionnaire

Thank you for considering your participation in our study called PERFORMD. We would like to fill out this questionnaire, so we can assess your eligibility.

First name/s: ……………………………………………………………………………………………………

Last name: ……………………………………………………………………………………………

Address: ……………………………………………………………………………………………

Postcode:…………………………………………………………………………………………

Phone number:……………………………………………………………………………………

E-mail address:……………………………………………………………………………………

Date of birth: ……………………………………………………………………………………

General Practitioner (GP)/Family doctor

Name:……………………………………………………………………………………………

Address:…………………………………………………………………………………………

Postcode:…………………………………………………………………………………………

Phone number:……………………………………………………………………………………

For peer review only - http://bmjopen.bmj.com/site/about/guidelines.xhtml

UNIVERSITY OF LEEDS

NHS
The Leeds
Teaching Hospitals
NHS Trust

Medical specialist (if applicable)

Name: …………………………………………………………………………………………….........

Address:……………………………………………………………………………………………........

Postcode: ………………………………………………………………………………….............

Phone number: ……………………………………………………………………………….........

1. What is your ethnic origin?
   ☐ White (Caucasian)
   ☐ Black (African-American)
   ☐ Asian
   ☐ Mixed

2. What is the highest educational qualification that you have?
   ☐ No qualifications
   ☐ Achieved GCSE grades D-G, NVQ Level 1, Skills For Life level 1, BTEC-award Certificate or diploma level 1, OCR National
   ☐ GCSE grades A*-C, NVQ Level 2, BTEC Award Certificate OR diploma level 2
   ☐ AS & A level, NVQ Level 3, Advanced Extension award, International Baccalaureate, OCR National
   ☐ NVQ Level 4, BTEC Professional award, Certificate of Higher Education
   ☐ BTEC Award Advanced professional / Bachelors Degree / Graduate Diploma
   ☐ University Masters Degree / Postgraduate diploma / NVQ Level 5 / BTEC Advanced Professional Award Certificate and Diploma level 7
   ☐ Doctorate (e.g. PhD, DClin.)

For peer review only

For peer review only - http://bmjopen.bmj.com/site/about/guidelines.xhtml

UNIVERSITY OF LEEDS

The Leeds Teaching Hospitals NHS Trust

3. What do you do?
   ☐ I am a student
   ☐ I am employed
   ☐ I am self employed
   ☐ I am a housewife, househusband
   ☐ I am unemployed

   ☐ I am unable to work (e.g. due to a disability)

   ☐ I am retired
   ☐ I do something else (e.g. volunteering), namely...

4. When is your expected due date?
   If you do not know exactly, try to estimate it as well as possible.

   |__|__| |__|__| |__|__|__|__|
   Day Month Year

5. How long have you been pregnant? (weeks)
   If you do not know exactly, try to estimate it as well as possible.

   ....

6. Do you have children?
   ☐ Yes, I have …. child(ren).
   ☐ No

7. Do you have a singleton pregnancy?
   ☐ Yes, I am expecting an single child
   ☐ No, I am expecting twins, triplets etc.

8. Would you say your general health is…..?
   ☐ Excellent
   ☐ Very Good
   ☐ Good
   ☐ Fair
   ☐ Poor
   ☐ Don't know/Not sure
   ☐ I rather not say

9. Are you diagnosed with diabetes (Type 1, Type 2, Gestational diabetes)?
   ☐ Yes, Please specify type of diabetes ....
   ☐ No

10. For how long have you been diagnosed with diabetes?
   If you do not know exactly, try to estimate it as well as possible.

For peer review only

For peer review only - http://bmjopen.bmj.com/site/about/guidelines.xhtml

UNIVERSITY OF LEEDS

The Leeds Teaching Hospitals NHS Trust

Please, specify in years ....

11. Did you take any medication (including diabetes medication) in the last month?
    ☐ No
    ☐ Yes

    If yes, please specify which medication, the dose and how many times a day.

    For example: Routine: Omeprazole 40mg once a day for 7 days or one-time treatment: amoxicillin 500mg once a for 7 days.

    ....

    ....

    ....

    ....

    ....

12. Do you currently smoke or use e-cigarettes?
    ☐ Yes - smoke cigarettes
    ☐ Yes - smoke cigars
    ☐ Yes - use e- cigarettes
    ☐ No, I quit smoking → go on to question 22
    ☐ No, I never smoked → go on to question 23

13. How many cigarettes/cigars do you normally smoke?
    ☐ 1-5 each day
    ☐ 6-10 each day
    ☐ More than 10 each day
    ☐ None, I smoke pipe or vape

14. What year did you start and quit smoking?
    Started: (yyyy) |__|__|__|__|
    Quit: (yyyy) |__|__|__|__|

For peer review only - http://bmjopen.bmj.com/site/about/guidelines.xhtml
For peer review only

UNIVERSITY OF LEEDS

NHS The Leeds Teaching Hospitals NHS Trust

15. How many units of alcohol did you normally (before pregnancy) consume during the week? (A Guide to the number of units of alcohol in some typical alcoholic drinks is provided). https://www.nhs.uk/Livewell/alcohol/Pages/alcohol-units.aspx

☐ I do not use any alcohol
☐ Less than 1 unit a week
☐ 1 - 5 units a week
☐ 6 - 7 units a week
☐ 8 - 15 units a week
☐ 16 - 30 units a week
☐ More than 30 units a week

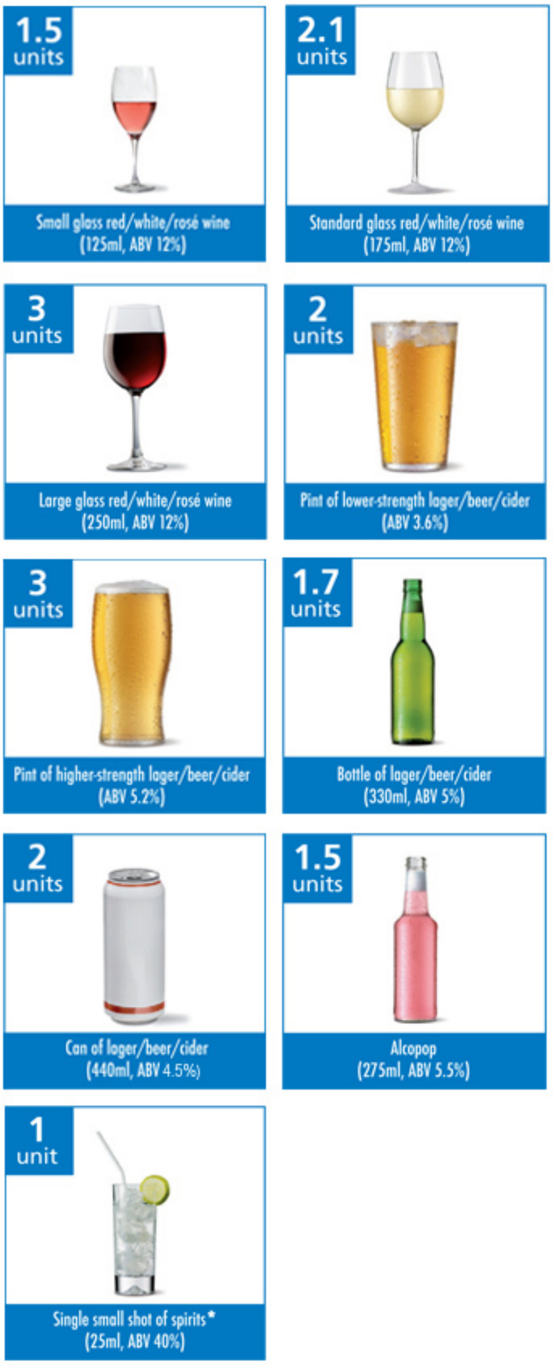

For peer review only - http://bmjopen.bmj.com/site/about/guidelines.xhtml

16. Do you have any food allergies?
☐ No
☐ Yes

If yes, please tick any allergies that apply below:

☐ Tree Nuts (e.g. walnuts, almonds, pine nuts, brazil nuts, and pecans)
☐ Peanuts
☐ Cow's milk
☐ Other milk
☐ Eggs
☐ Wheat
☐ Barley
☐ Oats
☐ Molluscs
☐ Lupin
☐ Sesame
☐ Sulphites
☐ Soy
☐ Mustard
☐ Celery
☐ Fish, shellfish and crustaceans
☐ Other, namely………………………………………………………………….

17. Do you use any dietary supplements? (i.e. vitamin supplements, minerals, fibres or probiotics). Examples of commonly used probiotics include:
    - Actimel drink
    - Activia yogurt
    - Benecol yogurt drink
    - Yakult drink
    - Arla Skyr yogurt drink
    - Creamier bio-live Irish yogurts
    - Probiotics from a local chemist or herbalist (e.g. Acidophilus Capsules)

☐ No
☐ Yes

If yes, what products have you used: How often do you use them (e.g. once per day):

…………………………………………… ……………………………………………

…………………………………………… ……………………………………………

…………………………………………… ……………………………………………

For peer review only - http://bmjopen.bmj.com/site/about/guidelines.xhtml
For peer review only

18. Do you have internet access on a computer, tablet or smartphone at home?
    ☐ Yes

    ☐ No

19. Are you currently participating in any other research studies?
    ☐ Yes
    ☐ No

    If yes, please specify which study and give brief explanation.

    ……………………………………………………………………………………………………

    ……………………………………………………………………………………………………

    ……………………………………………………………………………………………………

    ……………………………………………………………………………………………………

    ……………………………………………………………………………………………………

Can we keep this information on file and contact you about future studies? Yes / No

For peer review only

For peer review only - http://bmjopen.bmj.com/site/about/guidelines.xhtml

Suplementary section E: Medical history questionnaire

INFORMED

Individualised patieNt care and treatment FOR MatErnal Diabetes

Understanding the glycaemic profile of maternal diabetes using continuous glucose monitoring: intensive glucose profiling to inform patient care and treatment

For peer review only

Medical History Questionnaire

1. Do you currently have or have you had history of any of the following diseases?
If you have selected yes for any of the following conditions, please indicate which (if any) you are currently receiving treatment for? Please also state whether the condition limits you in your daily activities.

	Have you/ are you currently suffering from any of the following?		Are you receiving treatment for this disease?		Does this disease limit you in your daily activities?	
**Diseases**	*No*	*Yes*	*No*	*Yes*	*No*	*Yes*
Disease of heart/coronary arteries (angina pectoris, heart attack)						
Hypertension						
Lung disease						
Asthma						
Diabetes						
Stomach ulcers or other stomach disorders						
Kidney disease						
Liver disease						
Anaemia or any other blood disease						
Cancer						
Depression						
Eating disorder (e.g. anorexia/bulimia)						
Chronic fatigue syndrome (CFS)						

For peer review only - http://bmjopen.bmj.com/site/about/guidelines.xhtml

Stroke						
Gastrointestinal disease						
Oesophageal disease						
Thyroid disease						
High cholesterol						

If you are suffering from any other medical problems, please specify below:	*No*	*Yes*	*No*	*Yes*
………………………………………………………………………………………… ………………………………				
………………………………………………………………………………………… ………………………………				
………………………………………………………………………………………… ………………………………				

2. In the past, have you had any major abdominal surgery (please provide dates)?

Not had any major abdominal surgery in the past

Yes, had major abdominal surgery in the past, namely:

Laparoscopic (or key-hole): appendectomy Cholecystectomy

Open surgery: appendectomy Cholecystectomy

Other abdominal surgery, namely: ……………………………………….

Date/s of any surgery:………………………………………………………………………………

Appendectomy: removal of the appendix

Cholecystectomy: removal of the gall bladder

For peer review only - http://bmjopen.bmj.com/site/about/guidelines.xhtml

For peer review only

Suplementary section F: Modified Pregnancy Physical Activity Questionnaire (PPAQ)

INFORMED

Individualised patieNt care and treatment FOR MatErnal Diabetes

Understanding the glycaemic profile of maternal diabetes using continuous glucose monitoring: intensive glucose profiling to inform patient care and treatment

Physical activity questionnaire

During this trimester, how much time do you usually spend on:

1. Preparing meals (cook, set table, wash dishes)

None

Less than ½ hour per day

½ to almost 1 hour per day

1 to almost 2 hour per day

2 to almost 3 hour per day

3 or more hours per day

2. Taking care of an older adult

None

Less than ½ hour per day

½ to almost 1 hour per day

1 to almost 2 hour per day

2 to almost 3 hour per day

3 or more hours per day

3. Sitting and using a computer or writing, <u>not for work</u>

None

Less than ½ hour per day

For peer review only - http://bmjopen.bmj.com/site/about/guidelines.xhtml

½ to almost 1 hour per day

1 to almost 2 hour per day

2 to almost 3 hour per day

3 or more hours per day

4. Sitting at work or in class → If the participant does not work or study, skip to question 7.

None

Less than ½ hour per day

½ to almost 1 hour per day

1 to almost 2 hour per day

2 to almost 3 hour per day

3 or more hours per day

5. Standing or walking at work while carrying things (heavier than a 1 gallon milk jug)

None

Less than ½ hour per day

½ to almost 1 hour per day

1 to almost 2 hour per day

2 to almost 3 hour per day

3 or more hours per day

6. Standing or walking at work while not carrying anything

None

Less than ½ hour per day

½ to almost 1 hour per day

1 to almost 2 hour per day

2 to almost 3 hour per day

3 or more hours per day

7. Sitting and reading, talking or on the phone, not for work

None

For peer review only

For peer review only - http://bmjopen.bmj.com/site/about/guidelines.xhtml

Less than ½ hour per day

½ to almost 1 hour per day

1 to almost 2 hour per day

2 to almost 3 hour per day

3 or more hours per day

8. Watching TV or video

None

Less than ½ hour per day

½ to almost 1 hour per day

1 to almost 2 hour per day

2 to almost 3 hour per day

3 or more hours per day

9. Light cleaning (make beds, laundry, ironing, putting things away)

None

Less than ½ hour per day

½ to almost 1 hour per day

1 to almost 2 hour per day

2 to almost 3 hour per day

3 or more hours per day

10. Heavier cleaning (vacuum, mop, sweep, wash windows)

None

Less than ½ hour per day

½ to almost 1 hour per day

1 to almost 2 hour per day

2 to almost 3 hour per day

3 or more hours per day

11. Shopping (for food, clothes, or other)

For peer review only - http://bmjopen.bmj.com/site/about/guidelines.xhtml

None

Less than ½ hour per day

½ to almost 1 hour per day

1 to almost 2 hour per day

2 to almost 3 hour per day

3 or more hours per day

12. Gardening

None

Less than ½ hour per day

½ to almost 1 hour per day

1 to almost 2 hour per day

2 to almost 3 hour per day

3 or more hours per day

Please, fill out the next section if you have children. If you do not take care of children, you do not need to complete the this section.

During this trimester, taking care of children, how much time do you usually spend on:

13. Dressing, bathing, feeding children while you are <u>sitting</u>

None

Less than ½ hour per day

½ to almost 1 hour per day

1 to almost 2 hour per day

2 to almost 3 hour per day

3 or more hours per day

14. Dressing, bathing, feeding children while you are <u>standing</u>

None

Less than ½ hour per day

½ to almost 1 hour per day

For peer review only - http://bmjopen.bmj.com/site/about/guidelines.xhtml

1 to almost 2 hour per day

2 to almost 3 hour per day

3 or more hours per day

15. Playing with children while you are standing or sitting

None

Less than ½ hour per day

½ to almost 1 hour per day

1 to almost 2 hour per day

2 to almost 3 hour per day

3 or more hours per day

16. Playing with children while you are walking or running

None

Less than ½ hour per day

½ to almost 1 hour per day

1 to almost 2 hour per day

2 to almost 3 hour per day

3 or more hours per day

17. Carrying children

None

Less than ½ hour per day

½ to almost 1 hour per day

1 to almost 2 hour per day

2 to almost 3 hour per day

3 or more hours per day

During this trimester, how much time do you usually spend on exercising:

18. Walking to go places (such as to the bus, work, visiting). Not for fun or exercise

None

For peer review only

For peer review only - http://bmjopen.bmj.com/site/about/guidelines.xhtml

Less than ½ hour per day

½ to almost 1 hour per day

1 to almost 2 hour per day

2 to almost 3 hour per day

3 or more hours per day

19. Walking for fun or exercise

None

Less than ½ hour per day

½ to almost 1 hour per day

1 to almost 2 hour per day

2 to almost 3 hour per day

3 or more hours per day

20. Jogging

None

Less than ½ hour per day

½ to almost 1 hour per day

1 to almost 2 hour per day

2 to almost 3 hour per day

3 or more hours per day

21. Prenatal exercise class

None

Less than ½ hour per day

½ to almost 1 hour per day

1 to almost 2 hour per day

2 to almost 3 hour per day

3 or more hours per day

22. Doing other things for fun or exercise (such as swimming or dancing)?

For peer review only - http://bmjopen.bmj.com/site/about/guidelines.xhtml
For peer review only

None

Less than ½ hour per day

½ to almost 1 hour per day

1 to almost 2 hour per day

2 to almost 3 hour per day

3 or more hours per day

For peer review only

For peer review only - http://bmjopen.bmj.com/site/about/guidelines.xhtml

Suplementary section G: Modified Leeds Sleep Evaluation Questionnaire (LSEQ)

INFORMED

Individualised patieNt care and treatment FOR MatErnal Diabetes

Understanding the glycaemic profile of maternal diabetes using continuous glucose monitoring: intensive glucose profiling to inform patient care and treatment

Sleep quality questionnaire

How would you describe the way you currently fall asleep in comparison to usual?

1. More difficult than usual -------------- Easier than usual

2. Slower than usual -------------- More quickly than usual

3. I feel less sleepy than usual -------------- More sleepy than usual

How would you describe the quality of your sleep compared to normal sleep?

4. More restless than usual -------------- Calmer than usual

5. With more wakeful periods than usual ------------- With less wakeful periods

How would you describe your awakening in comparison to usual?

6. More difficult than usual -------------- Easier than usual

7. Requires a period of time longer than usual -------------- Shorter than usual

How do you feel when you wake up?

8. Tired -------------- Alert

How do you feel now?

9. Tired -------------- Alert

How would you describe your balance and co-ordination upon awakening?

10. More disrupted than usual -------------- Less disrupted than usual]

For peer review only

For peer review only - http://bmjopen.bmj.com/site/about/guidelines.xhtml

SPIRIT

STANDARD PROTOCOL ITEMS: RECOMMENDATIONS FOR INTERVENTIONAL TRIALS

SPIRIT 2013 Checklist: Recommended items to address in a clinical trial protocol and related documents*

Section/item	Item No	Description	Pg
**Administrative information**			
Title	1	Descriptive title identifying the study design, population, interventions, and, if applicable, trial acronym	X
Trial registration	2a	Trial identifier and registry name. If not yet registered, name of intended registry	X
	2b	All items from the World Health Organization Trial Registration Data Set	X
Protocol version	3	Date and version identifier	X
Funding	4	Sources and types of financial, material, and other support	X
Roles and responsibilities	5a	Names, affiliations, and roles of protocol contributors	X
	5b	Name and contact information for the trial sponsor	X
	5c	Role of study sponsor and funders, if any, in study design; collection, management, analysis, and interpretation of data; writing of the report; and the decision to submit the report for publication, including whether they will have ultimate authority over any of these activities	X
	5d	Composition, roles, and responsibilities of the coordinating centre, steering committee, endpoint adjudication committee, data management team, and other individuals or groups overseeing the trial, if applicable (see Item 21a for data monitoring committee)	X
**Introduction**			
Background and rationale	6a	Description of research question and justification for undertaking the trial, including summary of relevant studies (published and unpublished) examining benefits and harms for each intervention	X
	6b	Explanation for choice of comparators	X
Objectives	7	Specific objectives or hypotheses	X

For peer review only

Trial design	8	Description of trial design including type of trial (eg, parallel group, crossover, factorial, single group), allocation ratio, and framework (eg, superiority, equivalence, noninferiority, exploratory)	X
**Methods: Participants, interventions, and outcomes**			
Study setting	9	Description of study settings (eg, community clinic, academic hospital) and list of countries where data will be collected. Reference to where list of study sites can be obtained	X
Eligibility criteria	10	Inclusion and exclusion criteria for participants. If applicable, eligibility criteria for study centres and individuals who will perform the interventions (eg, surgeons, psychotherapists)	X
Interventions	11a	Interventions for each group with sufficient detail to allow replication, including how and when they will be administered	X
	11b	Criteria for discontinuing or modifying allocated interventions for a given trial participant (eg, drug dose change in response to harms, participant request, or improving/worsening disease)	X
	11c	Strategies to improve adherence to intervention protocols, and any procedures for monitoring adherence (eg, drug tablet return, laboratory tests)	X
	11d	Relevant concomitant care and interventions that are permitted or prohibited during the trial	X
Outcomes	12	Primary, secondary, and other outcomes, including the specific measurement variable (eg, systolic blood pressure), analysis metric (eg, change from baseline, final value, time to event), method of aggregation (eg, median, proportion), and time point for each outcome. Explanation of the clinical relevance of chosen efficacy and harm outcomes is strongly recommended	X
Participant timeline	13	Time schedule of enrolment, interventions (including any run-ins and washouts), assessments, and visits for participants. A schematic diagram is highly recommended (see Figure)	X
Sample size	14	Estimated number of participants needed to achieve study objectives and how it was determined, including clinical and statistical assumptions supporting any sample size calculations	X

For peer review only - http://bmjopen.bmj.com/site/about/guidelines.xhtml

Recruitment	15	Strategies for achieving adequate participant enrolment to reach target sample size	X

Methods: Assignment of interventions (for controlled trials)

Allocation:

Sequence generation	16a	Method of generating the allocation sequence (eg, computer-generated random numbers), and list of any factors for stratification. To reduce predictability of a random sequence, details of any planned restriction (eg, blocking) should be provided in a separate document that is unavailable to those who enrol participants or assign interventions	X
Allocation concealment mechanism	16b	Mechanism of implementing the allocation sequence (eg, central telephone; sequentially numbered, opaque, sealed envelopes), describing any steps to conceal the sequence until interventions are assigned	X
Implementation	16c	Who will generate the allocation sequence, who will enrol participants, and who will assign participants to interventions	X
Blinding (masking)	17a	Who will be blinded after assignment to interventions (eg, trial participants, care providers, outcome assessors, data analysts), and how	X
	17b	If blinded, circumstances under which unblinding is permissible, and procedure for revealing a participant's allocated intervention during the trial	X

Methods: Data collection, management, and analysis

Data collection methods	18a	Plans for assessment and collection of outcome, baseline, and other trial data, including any related processes to promote data quality (eg, duplicate measurements, training of assessors) and a description of study instruments (eg, questionnaires, laboratory tests) along with their reliability and validity, if known. Reference to where data collection forms can be found, if not in the protocol	X
	18b	Plans to promote participant retention and complete follow-up, including list of any outcome data to be collected for participants who discontinue or deviate from intervention protocols	X
Data management	19	Plans for data entry, coding, security, and storage, including any related processes to promote data quality (eg, double data entry; range checks for data values). Reference to where details of data management procedures can be found, if not in the protocol	X

For peer review only - http://bmjopen.bmj.com/site/about/guidelines.xhtml

Statistical methods	20a	Statistical methods for analysing primary and secondary outcomes. Reference to where other details of the statistical analysis plan can be found, if not in the protocol	X
	20b	Methods for any additional analyses (eg, subgroup and adjusted analyses)	X
	20c	Definition of analysis population relating to protocol non-adherence (eg, as randomised analysis), and any statistical methods to handle missing data (eg, multiple imputation)	X
**Methods: Monitoring**			
Data monitoring	21a	Composition of data monitoring committee (DMC); summary of its role and reporting structure; statement of whether it is independent from the sponsor and competing interests; and reference to where further details about its charter can be found, if not in the protocol. Alternatively, an explanation of why a DMC is not needed	X
	21b	Description of any interim analyses and stopping guidelines, including who will have access to these interim results and make the final decision to terminate the trial	X
Harms	22	Plans for collecting, assessing, reporting, and managing solicited and spontaneously reported adverse events and other unintended effects of trial interventions or trial conduct	X
Auditing	23	Frequency and procedures for auditing trial conduct, if any, and whether the process will be independent from investigators and the sponsor	X
**Ethics and dissemination**			
Research ethics approval	24	Plans for seeking research ethics committee/institutional review board (REC/IRB) approval	X
Protocol amendments	25	Plans for communicating important protocol modifications (eg, changes to eligibility criteria, outcomes, analyses) to relevant parties (eg, investigators, REC/IRBs, trial participants, trial registries, journals, regulators)	X
Consent or assent	26a	Who will obtain informed consent or assent from potential trial participants or authorised surrogates, and how (see Item 32)	X
	26b	Additional consent provisions for collection and use of participant data and biological specimens in ancillary studies, if applicable	X

w only

Confidentiality	27	How personal information about potential and enrolled participants will be collected, shared, and maintained in order to protect confidentiality before, during, and after the trial	X
Declaration of interests	28	Financial and other competing interests for principal investigators for the overall trial and each study site	X
Access to data	29	Statement of who will have access to the final trial dataset, and disclosure of contractual agreements that limit such access for investigators	X
Ancillary and post-trial care	30	Provisions, if any, for ancillary and post-trial care, and for compensation to those who suffer harm from trial participation	X
Dissemination policy	31a	Plans for investigators and sponsor to communicate trial results to participants, healthcare professionals, the public, and other relevant groups (eg, via publication, reporting in results databases, or other data sharing arrangements), including any publication restrictions	X
	31b	Authorship eligibility guidelines and any intended use of professional writers	X
	31c	Plans, if any, for granting public access to the full protocol, participant-level dataset, and statistical code	X
**Appendices**			
Informed consent materials	32	Model consent form and other related documentation given to participants and authorised surrogates	X
Biological specimens	33	Plans for collection, laboratory evaluation, and storage of biological specimens for genetic or molecular analysis in the current trial and for future use in ancillary studies, if applicable	X

*It is strongly recommended that this checklist be read in conjunction with the SPIRIT 2013 Explanation & Elaboration for important clarification on the items. Amendments to the protocol should be tracked and dated. The SPIRIT checklist is copyrighted by the SPIRIT Group under the Creative Commons "Attribution-NonCommercial-NoDerivs 3.0 Unported" license.

For peer review only
